# The Mevalonate Pathway Is Indispensable for Adipocyte Survival

**DOI:** 10.1016/j.isci.2018.10.019

**Published:** 2018-10-21

**Authors:** Yu-Sheng Yeh, Huei-Fen Jheng, Mari Iwase, Minji Kim, Shinsuke Mohri, Jungin Kwon, Satoko Kawarasaki, Yongjia Li, Haruya Takahashi, Takeshi Ara, Wataru Nomura, Teruo Kawada, Tsuyoshi Goto

**Affiliations:** 1 Laboratory of Molecular Function of Food, Division of Food Science and Biotechnology, Graduate School of Agriculture, Kyoto University, Uji, Kyoto, Japan; 2 Research Unit for Physiological Chemistry, Center for the Promotion of Interdisciplinary Education and Research, Kyoto University, Kyoto, Japan

**Keywords:** Pathophysiology, Molecular Mechanism of Behavior, Diabetology, Specialized Functions of Cells

## Abstract

The mevalonate pathway is essential for the synthesis of isoprenoids and cholesterol. Adipose tissue is known as a major site for cholesterol storage; however, the role of the local mevalonate pathway and its synthesized isoprenoids remains unclear. In this study, adipose-specific mevalonate pathway-disrupted (aKO) mice were generated through knockout of 3-hydroxy-3-methylglutaryl-CoA (HMG-CoA) reductase (HMGCR). aKO mice showed serious lipodystrophy accompanied with glucose and lipid metabolic disorders and hepatomegaly. These metabolic variations in aKO mice were dramatically reversed after fat transplantation. In addition, HMGCR-disrupted adipocytes exhibited loss of lipid accumulation and an increase of cell death, which were ameliorated by the supplementation of mevalonate and geranylgeranyl pyrophosphate but not farnesyl pyrophosphate and squalene. Finally, we found that apoptosis may be involved in adipocyte death induced by HMGCR down-regulation. Our findings indicate that the mevalonate pathway is essential for adipocytes and further suggest that this pathway is an important regulator of adipocyte turnover.

## Highlights

• HMGCR deficiency in adipocytes causes lipodystrophy
• HMGCR deficiency in adipocytes triggers several metabolic disorders
• Cholesterol and pioglitazone cannot improve HMGCR-deficiency-induced lipodystrophy
• GGPP is critical for adipocyte survival by regulating apoptosis

## Graphical Abstract

**Figure:**
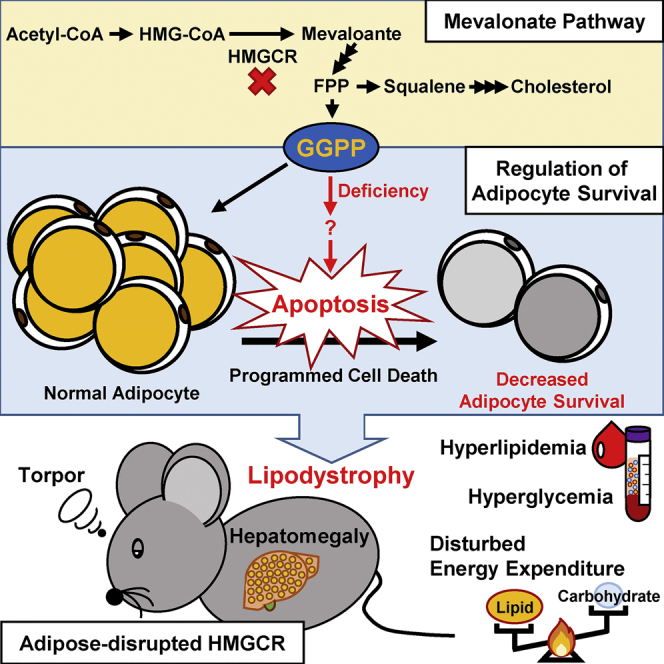

## Introduction

The mevalonate (MVA) pathway produces sterols, especially cholesterol, which is essential for cell membrane structure, bile acids, and steroid hormones (Goldstein and Brown, 1990). Moreover, the MVA pathway is also known to synthesize isoprenoids, such as farnesyl pyrophosphate (FPP) and geranylgeranyl pyrophosphate (GGPP), which are essential sources of protein prenylation (Casey, 1992). In particular, prenylation comprises a post-translational process that modifies a multitude of proteins involved in protein synthesis and glycosylation, intracellular signaling, gene expression, and cell growth (Casey, 1992, Goldstein and Brown, 1990). In addition, 3-hydroxy-3-methylglutaryl-CoA (HMG-CoA) reductase (HMGCR), a rate-limiting enzyme in the MVA pathway, catalytically converts HMG-CoA to MVA and is further repressed via negative feedback regulation (Goldstein and Brown, 1990).

As the MVA pathway affects various metabolic pathways, inhibitors of HMGCR, also known as statins, have been extensively used in the clinic as an effective treatment for hypercholesterolemia, in addition to being considered as potential treatments for certain features of cardiovascular disease and hepatic disorders (Kamal et al., 2017, Wadhera et al., 2016). However, accumulating data indicate that the usage of statins has certain unexpected cholesterol-independent pleiotropic effects, such as increased risk of new-onset diabetes or muscle disorders (Thompson et al., 2003, Zaharan et al., 2013). Skeletal muscle-specific HMGCR knockout mice also exhibited similar symptoms (Osaki et al., 2015). Furthermore, disruption of HMGCR in the liver, the major target organ for statin metabolism, has been reported to cause liver steatosis and death (Nagashima et al., 2012). These phenomena are thought to be caused by insufficient intermediate products generated in the MVA pathway (Bang and Okin, 2014, Brault et al., 2014) and suggest that HMGCR is important for mammals and plays a different role in each organ.

Obesity, defined as an increase in adipose tissue mass, is thought to be highly related to lipid metabolism diseases, such as hypercholesterolemia (Fasshauer and Bluher, 2015, Lackey and Olefsky, 2016). In addition, adipose tissue is known to be the major organ for cholesterol storage, thus serving an important function in regulating systemic cholesterol metabolism (Bays et al., 2013, Krause and Hartman, 1984). However, the role of the MVA pathway in adipose tissue remains completely unknown. Previous studies have shown that MVA-derived metabolites, such as FPP and GGPP, serve as endogenous regulators of adipocyte function (Goto et al., 2011, Yeh et al., 2016). Moreover, MVA pathway-related gene expression is partially enhanced in the adipose tissue of an obese/diabetic mouse model (Yeh et al., 2016, Yu et al., 2011). These results reveal that isoprenoids may play an important role in the regulation of adipose tissue function. Thus, to further clarify the detailed role of isoprenoids in adipose tissue function, we generated adipose-specific MVA pathway-disrupted (aKO) mice through specific knockout of HMGCR in adipose tissue. Here, we show that the MVA pathway in adipocytes is both necessary and sufficient to maintain adipocyte survival, which in turn further influences systemic homeostasis.

## Results

### aKO Mice Show Systemic Lipodystrophy

**Figure 1:**
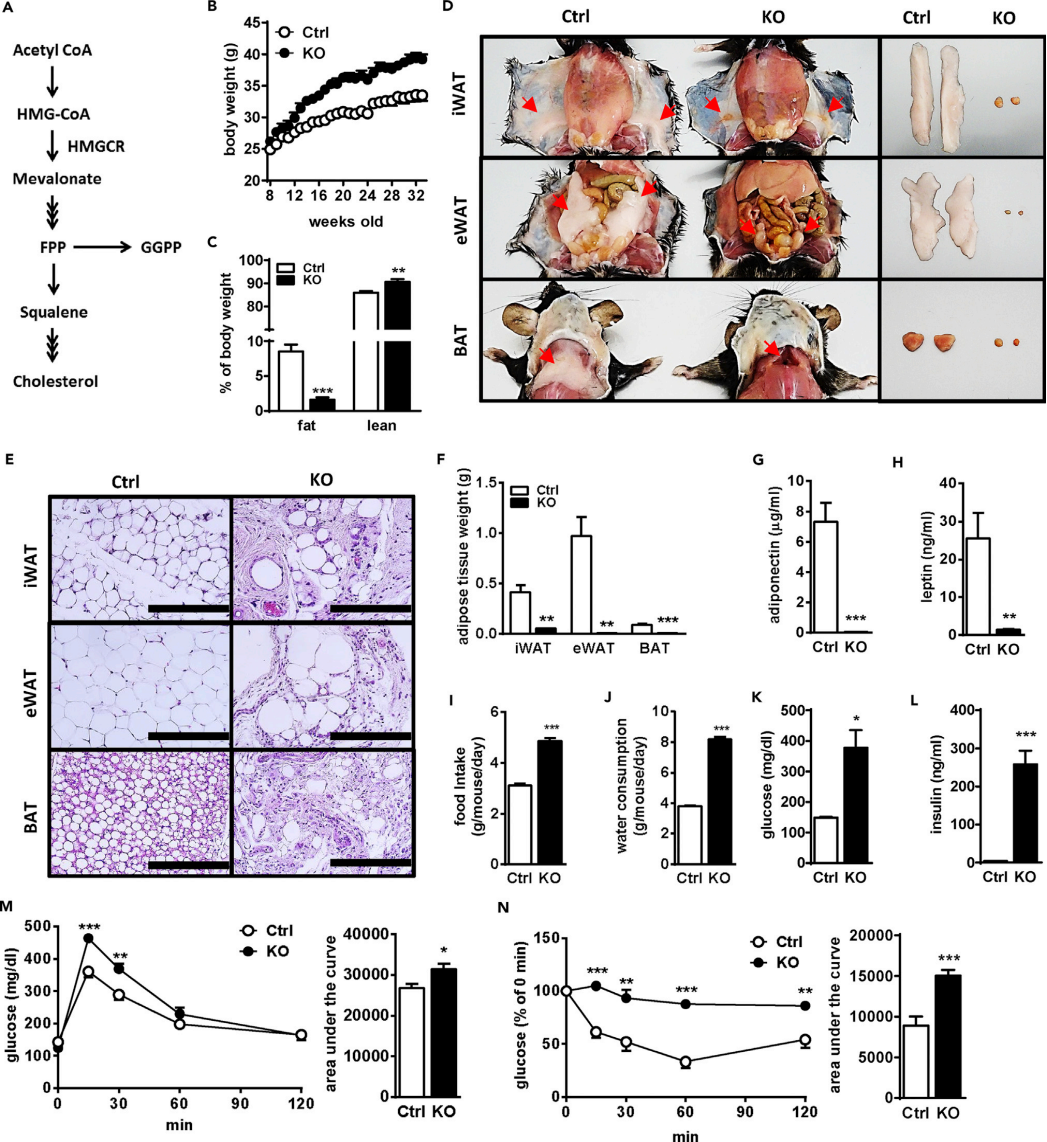
aKO Mice Exhibit Lipodystrophy and Diabetic Symptoms (A) Schematic illustration of the mevalonate pathway. (B) Body weight change of control (Ctrl) and aKO mice. (C) Relative fat and lean mass levels of 15-week-old Ctrl and aKO mice. (D) Gross morphology of iWAT (top panel), eWAT (also the liver, middle panel), and BAT (bottom panel) of Ctrl and aKO mice at 32 weeks of age. The arrows indicate the tissue position. (E) H&E-stained tissue sections of iWAT (top panel), eWAT (middle panel), and BAT (bottom panel) from Ctrl and aKO mice at 32 weeks of age. Scale bars, 200 μm. (F) Adipose tissue weights of Ctrl and aKO mice at 32 weeks of age. (G and H) Plasma adiponectin (G) and leptin (H) levels in Ctrl and aKO mice quantified at 32 weeks of age. (I and J) Food intake (I) and water consumption (J) of Ctrl and aKO mice measured from 14 to 32 weeks of age. (K and L) Plasma glucose (K) and insulin (L) levels analyzed in 32-week-old Ctrl and aKO mice. (M) GTT in Ctrl and aKO mice was assessed at 16 weeks of age. (N) ITT in Ctrl and aKO mice was assessed at 18 weeks of age. All mice were male and fed a normal diet (ND). Bars represent the mean ± SE. (n = 6–8). Significant differences were determined by Student's t test compared with Ctrl: *p < 0.05, **p < 0.01, ***p < 0.001. See also Figure S2.

To study the role of the MVA pathway (Figure 1A) in adipose tissues, we generated aKO mice using the Cre-loxP system. The floxed HMGCR mice, designed as shown in Figure S1A, were crossed with adiponectin-Cre mice (Eguchi et al., 2011). HMGCR knockout alleles were observed only in the genomic DNA extracted from white adipose tissue (WAT) and brown adipocyte tissue (BAT) and not in the DNA from other tissues in heterozygous aKO mice (Figure S1B).

**Figure 2:**
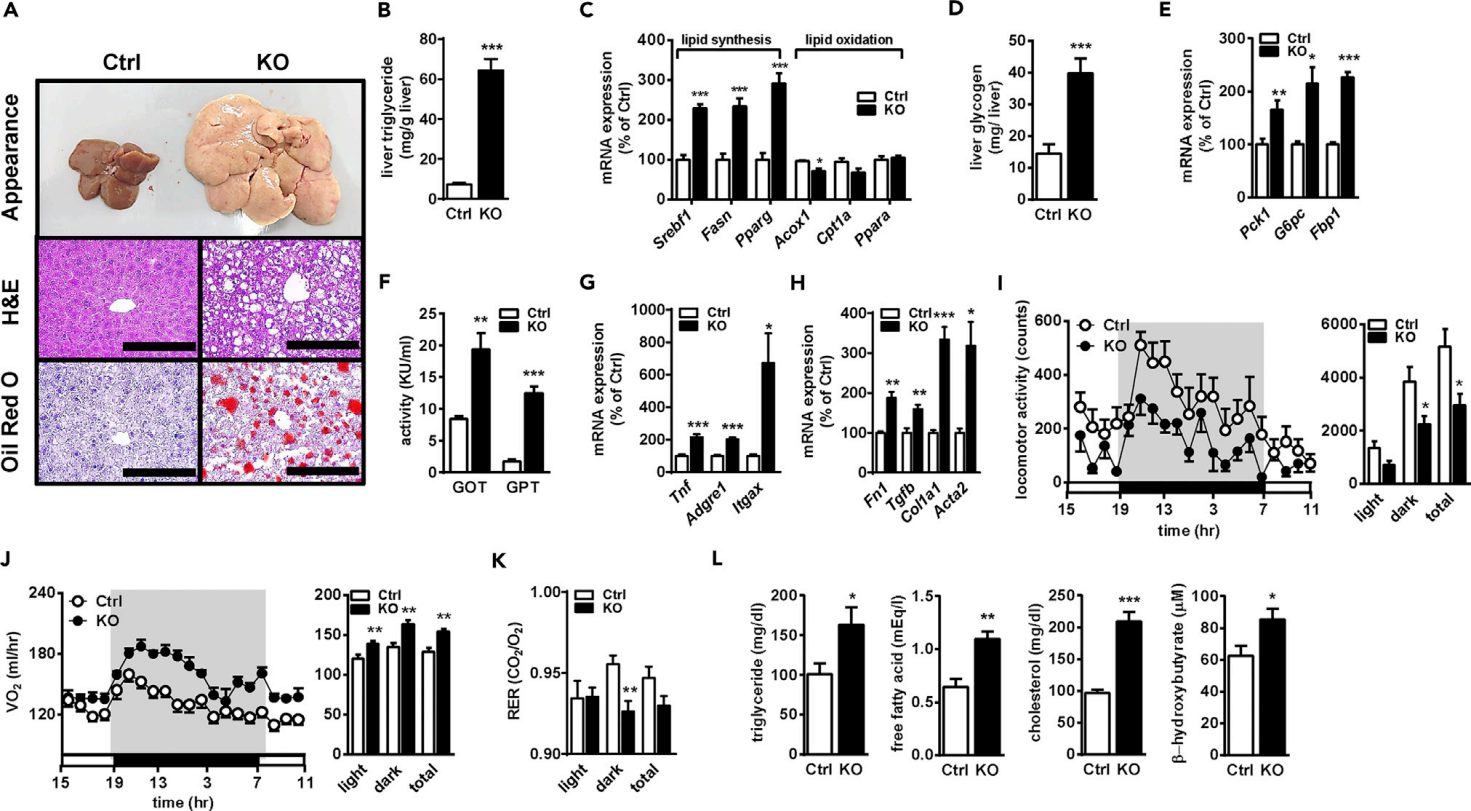
Impaired Liver Function and Metabolic Homeostasis in aKO Mice (A) Gross morphology of the liver (top panel) and liver sections stained with H&E (middle panel) and oil red O (bottom panel) in control (Ctrl) and aKO mice at 32 weeks of age. Scale bars, 200 μm. (B and C) Hepatic TG accumulation (B) and lipid metabolism-related gene expression (C) levels in 32-week-old Ctrl and aKO mice. (D and E) Hepatic glycogen (D) and gluconeogenesis-related gene expression (E) in 32-week-old Ctrl and aKO mice. (F–H) Plasma GOT and GPT activity (F) and hepatic inflammation- (G) and fibrosis- (H) related gene expression in 32-week-old Ctrl and aKO mice. (I–K) Locomotor activity (I), oxygen consumption (J), and RER (K) in 20- to 23-week-old Ctrl and aKO mice. (L) Plasma TG, FFA, cholesterol, and β-HB levels in 32-week-old Ctrl and aKO mice. All mice were male and fed a normal diet (ND). Values are presented as mean ± SE. (n = 6–8). Significant differences were determined by Student's t test compared with Ctrl: *p < 0.05, **p < 0.01, ***p < 0.001. See also Figure S2.

**Figure 3:**
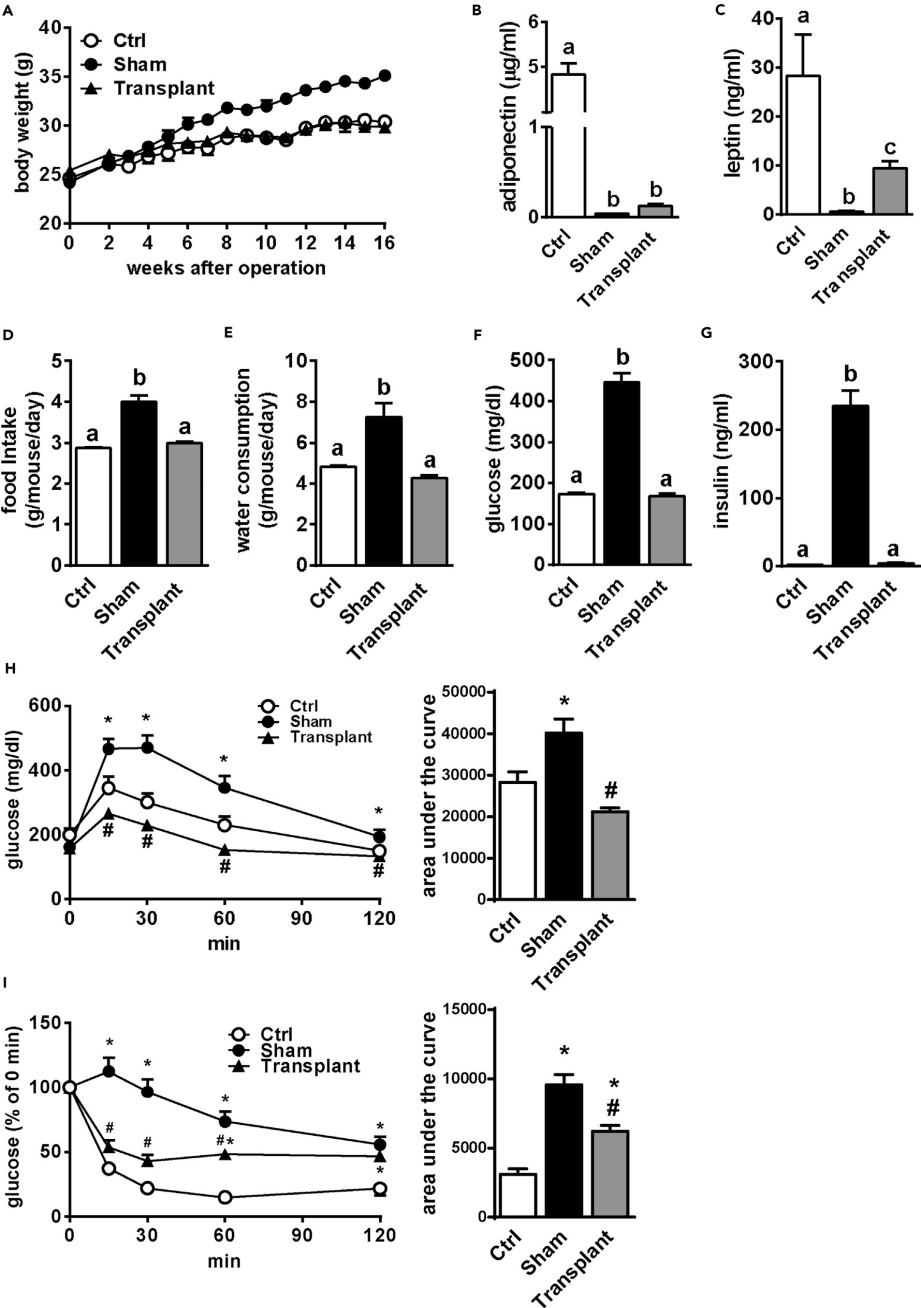
Implantation of WAT Reverses Diabetic Consequences in aKO Mice (A) Body weight change of sham-operated and WAT-transplanted aKO and control (Ctrl) mice as measured for 16 weeks. (B and C) Plasma adiponectin (B) and leptin (C) levels at 16 weeks after surgery. (D–G) Food intake (D) and water consumption (E), and plasma glucose (F) and insulin (G) levels as measured from 2 to 14 weeks after surgery. Bars represent mean ± SE. (n = 6–8). Bars with different letters represent significant difference (p < 0.05) by one-way ANOVA with a post-hoc Tukey HSD test (C–H). (H) GTT was performed at 6 weeks after transplantation. (I) ITT was performed at 8 weeks after transplantation. Bars are presented as mean ± SE. (n = 6–8). Means with * (Sham and Transplant versus Ctrl) and # (Transplant versus Sham) presented significant differences (p < 0.05) by one-way ANOVA with post-hoc Tukey HSD test.

Eight-week-old male (Figures 1, 2, and 3) and female mice (Figure S2) were used. The body weights of aKO mice of both genders were higher than those of control (Ctrl) mice (Figures 1B and S2A). Whole-body MRI scanning showed that fat mass was drastically decreased, whereas lean mass was slightly increased in aKO mice (Figures 1C and S2B). As shown in Figure 1D, we observed that aKO mice lost almost all fat pads, including inguinal WAT (iWAT), epididymal WAT (eWAT), and BAT. Furthermore, compared with Ctrl mice, a very limited number of lipid-laden adipocytes was observed in adipose tissues (iWAT, eWAT, and BAT) in aKO mice (Figure 1E). Consistent with the appearance of WAT and BAT, the tissue weight and levels of plasma adipokines including adiponectin and leptin were extremely reduced in aKO mice (Figures 1F–1H and S2C–S2E). Although the consumption of a high-fat diet (HFD) reversed the difference in body weight gain between the two genotype groups (Figure S3A), this appeared to mainly result from increases of fat weight in the Ctrl group rather than from lipodystrophy amelioration in the aKO group (Figures S3B–S3E). These results suggest that HMGCR in the adipose tissue plays a critical role in the maintenance of adipose mass and adipokine levels.

### aKO Mice Exhibit Severe Diabetes

Food intake and water consumption in aKO mice were obviously higher than those in Ctrl mice (Figures 1I, 1J, S2F, and S2G). Moreover, the non-fasting plasma glucose and insulin levels were markedly enhanced in aKO mice in both the normal diet (ND) (Figures 1K, 1L, S2H, and S2I) and HFD (Figures S3F and S3G) conditions, suggesting that glucose metabolism was disrupted in aKO mice. The glucose tolerance test (GTT) showed that plasma glucose levels were significantly higher in aKO mice after oral glucose administration, and the area under the curve also showed that glucose tolerance was reduced in aKO mice (Figure 1M). In the insulin tolerance test (ITT), plasma glucose levels were effectively reduced after insulin injection in Ctrl mice but not in aKO mice (Figure 1N). Female mice also showed similar results in GTT and ITT (Figures S2J and S2K). These results clearly show that ablation of HMGCR in adipose tissues induced lipodystrophy accompanied by severe dysfunction of glucose metabolism.

### aKO Mice Develop Ectopic Lipid Deposition in the Liver

Both male and female aKO mice had enlarged liver (Figures 2A and S2L, Table S2). Moreover, many bubble-like areas were found in liver sections of aKO mice stained with hematoxylin and eosin (H&E) (Figure 2A). Oil red O staining indicated obviously increased lipid droplets in aKO compared with Ctrl mice (Figure 2A). Consistent with this finding, hepatic triglyceride (TG) accumulation in aKO mice was enhanced (Figures 2B and S2M). As shown in Figure 2C, hepatic lipid-synthesis-related genes, such as sterol regulatory element-binding protein 1 (*Srebf1*), fatty acid synthase (*Fasn*), and peroxisome proliferator-activated receptor gamma (*Pparg*), were highly expressed in aKO compared with Ctrl mice, whereas very limited changes were observed in lipid-oxidation-related gene expression, including that of acyl-coenzyme A oxidase 1 (*Acox1*), carnitine palmitoyltransferase IA (*Cpt1a*), and peroxisome proliferator-activated receptor alpha (*Ppara*). These results suggest that increased hepatic lipid accumulation in aKO mice might mainly result from the up-regulation of lipid biosynthesis. In addition, hepatic liver glycogen showed a 2.5-fold increase in aKO mice compared with Ctrl mice, and hepatic gluconeogenesis-related gene expression, such as that of phosphoenolpyruvate carboxykinase (*Pck1*), glucose-6-phosphatase (*G6pc*), and fructose-1,6-bisphosphate (*Fbp1*), was also higher in aKO compared with Ctrl mice (Figures 2D and 2E). These data suggest that adipose-disrupted HMGCR possibly affected hepatic glucose metabolism.

The activities of plasma glutamic-oxaloacetic transaminase (GOT) and glutamic-pyruvic transaminase (GPT), the main hepatic injury markers, were significantly elevated in aKO mice compared with Ctrl mice (Figures 2F and S2N). To determine whether aKO mice exhibited progressive hepatic fibrosis and inflammation, associated gene expression levels were evaluated. The expression of inflammatory marker genes, such as tumor necrosis factor alpha (*Tnf*), EGF-like module-containing mucin-like hormone receptor-like 1 (*Adgre1*, also known as F4/80), and integrin, alpha X (*Itgax*, also known as CD11c), was increased in aKO mice (Figure 2G). Moreover, expression of fibrosis-related genes including fibronectin (*Fn1*); transforming growth factor β (*Tgfb*); collagen, type I, alpha 1 (*Col1a1*); and alpha-actin-2 (*Acta2*, also known as alpha smooth muscle actin) was also increased in the liver of aKO mice (Figure 2H). These results indicate that HMGCR ablation in adipose tissue induced abnormal liver function with potential progression toward cirrhosis.

### Adipose-Specific Disruption of HMGCR Alters the Pattern of Energy Expenditure

Although locomotor activity was significantly reduced in aKO mice (Figures 2I and S2O), the oxygen consumption rate was increased compared with Ctrl mice (Figures 2J and S2P). The respiratory exchange ratio (RER) was decreased in aKO mice in the dark state, suggesting that aKO mice prefer to use fatty acid as an energy source compared with Ctrl mice (Figures 2K and S2Q). Consistent with the RER results, we found that plasma β-hydroxybutyric acid (β-HB), a type of ketone body metabolized from fatty acid, was up-regulated, accompanied by an increase of several lipids, such as TG, free fatty acid (FFA), and cholesterol (Figures 3L and S3R). Thus, these data indicate that adipose-specific HMGCR ablation disrupts lipid and energy metabolism, as well as locomotor activity.

### Thiazolidinedione Improves Glucose-Metabolic Disorders without Rescuing Adipose Tissue Mass in aKO Mice

A previous study reported that MVA metabolites could modulate PPARγ activity, which plays a central role in adipocyte differentiation (Goto et al., 2011). Therefore, to examine whether lipodystrophy in aKO mice resulted from a reduction of PPARγ activity, we next orally administered a synthetic PPARγ agonist (pioglitazone [Pio], 30 mg/kg/day) to the mice for 10 weeks. Although Pio treatment reduced body weight gain and enhanced fat mass in aKO mice, the lack of adipose tissue in aKO mice was not improved (Figures S4A–S4C). Furthermore, Pio treatment further aggravated hepatomegaly, hepatic TG accumulation, and enhanced plasma GOT and GPT activities in aKO mice (Figure S4D). Pio treatment increased the plasma adiponectin level and decreased the plasma leptin level in Ctrl mice but had no such effects in aKO mice (Figures S4E and S4F). Although Pio failed to rescue the fat mass, the hyperphagia, hyperdipsia, hyperglycemia, and hyperinsulinemia were ameliorated by Pio treatment in aKO mice (Figures S4G–S4J). Taken together, these results indicate that PPARγ agonist treatment improved several metabolic disorders independently of adipose tissue function in aKO mice, suggesting that HMGCR-mediated lipodystrophy was minimally associated with the deficiency of MVA-derivative PPARγ ligands.

### Implantation of Adipose Tissue Reverses Diabetic Consequences in aKO Mice

Next, to investigate whether metabolic abnormalities in aKO mice were related to lipodystrophy, we performed WAT implantation. At 16 weeks after implantation, the fat grafts were successfully implanted subcutaneously and their weight was enhanced by approximately 1.8-fold compared with the initially transplanted fat weight (Figure S5A). The transplanted fat pads exhibited blood vessel formation and showed normal histology (Figures S5B and S5C). At six weeks after surgery, sham-operated (Sham) mice were significantly heavier than Ctrl and transplanted (Transplant) mice, which showed no significant difference in body weight (Figure 3A). Notably, however, fat implantation only resulted in mild recovery in plasma leptin and adiponectin levels (Figures 3B and 3C). Furthermore, we found that the enhanced food intake and water consumption in Sham mice were completely reversed by fat transplantation (Figures 3D and 3E). In addition, glucose metabolic disturbances including hyperglycemia, glucose intolerance, and insulin resistance in aKO mice were almost entirely reversed by fat transplantation (Figures 3F–3I). These results demonstrated that the severe diabetic symptoms occurring in adipose-specific HMGCR knockout were mainly due to WAT deficiency.

### Adipose Tissue Implantation Reverses Defects of Hepatic Metabolism in aKO Mice

**Figure 4:**
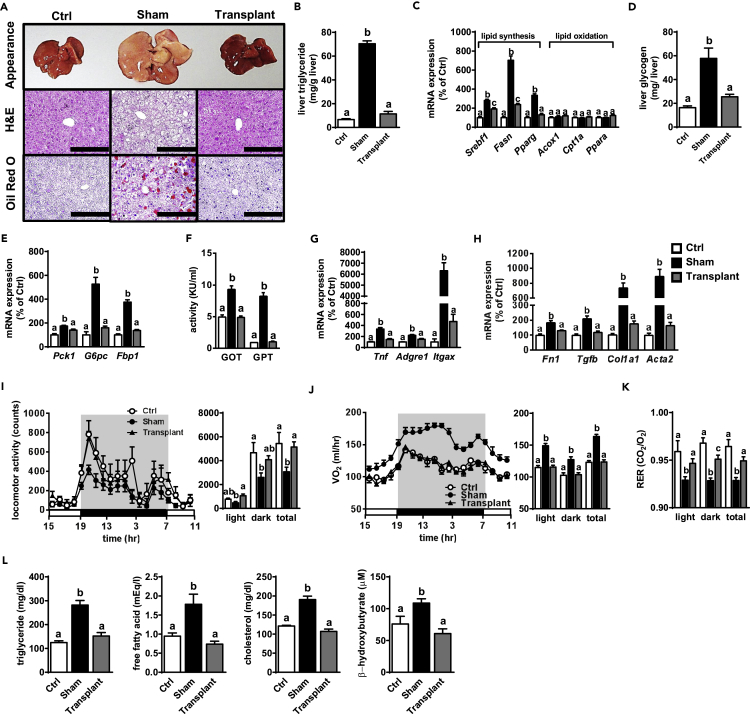
Hepatic Function and Systemic Metabolism Are Improved in Fat-Transplanted aKO Mice (A) Gross morphology of the liver (top panel) and liver sections stained with H&E (middle panel) and oil red O (bottom panel) at 16 weeks after surgery. Scale bars, 200 μm. (B and C) Hepatic TG accumulation (B) and lipid metabolism-related gene expression (C) at 16 weeks after surgery. (D and E) Hepatic glycogen (D) and gluconeogenesis-related gene expression (E) at 16 weeks after surgery. (F–H) Plasma GOT and GPT activity (F), and hepatic inflammation- (G) and fibrosis- (H) related gene expression at 16 weeks after surgery. (I–K) Locomotor activity (I), oxygen consumption (J), and RER (K) at 16 weeks after surgery. (L) Plasma TG, FFA, cholesterol, and β-HB levels at 16 weeks after surgery. Bars represent mean ± SE. (n = 6–8). Bars with different letters represent significant difference (p < 0.05) by one-way ANOVA with a post-hoc Tukey HSD test.

We next addressed whether the defective hepatic phenomena in aKO mice could be improved through fat transplantation. As shown in Figure 4A, the abnormal liver appearance and hepatic histology in Sham mice were reversed in Transplant mice. Consistent with this finding, hepatic TG accumulation levels were normalized by fat transplantation in aKO mice (Figure 4B). The gene expression associated with lipid synthesis was significantly up-regulated in the Sham compared with the Ctrl group, whereas such up-regulation was largely diminished in the Transplant group (Figure 4C). Furthermore, fat implantation also reversed the abnormalities in both total hepatic glycogen levels and gluconeogenesis-related gene expression in aKO mice (Figures 4D and 4E). These results suggest that both abnormal hepatic lipid and glucose metabolism in aKO mice mainly resulted from insufficient WAT.

Consistent with liver appearance and morphological variation, the increased plasma GOT and GPT activities in the Sham group were both reduced to the levels in the Ctrl group by fat transplantation (Figure 4F). Moreover, inflammation- and fibrosis-related gene expression were increased in the Sham group, whereas these changes were significantly reversed in the Transplant group (Figures 4G and 4H). These results suggest that fat implantation likely improved hepatic inflammation and fibrosis in aKO mice.

### Fat Transplantation Partially Improves the Pattern of Energy Expenditure in aKO Mice

Notably, the decreased locomotor activity was completely reversed by fat implantation in aKO mice (Figure 4I). As shown in Figure 4J, Sham mice showed higher oxygen consumption than Ctrl mice, whereas this enhancement was depressed in Transplant mice. Furthermore, based on the results of RER, the energy source was shifted from lipid to carbohydrate usage in aKO mice by fat transplantation (Figure 4K). Consistent with this finding, the increase in plasma β-HB level was reversed by fat implantation in aKO mice as well (Figure 4L), indicating that the disrupted lipid metabolism in aKO mice was possibly improved by fat transplantation. As expected, adipose tissue implantation reversed the up-regulation of plasma lipid levels in aKO mice (Figure 4L). These findings support that the disrupted metabolism and activity pattern in aKO mice might be largely due to an insufficiency of systemic adipose tissue.

### Inadequate HMGCR Causes Adipocyte Death *In Vitro*

**Figure 5:**
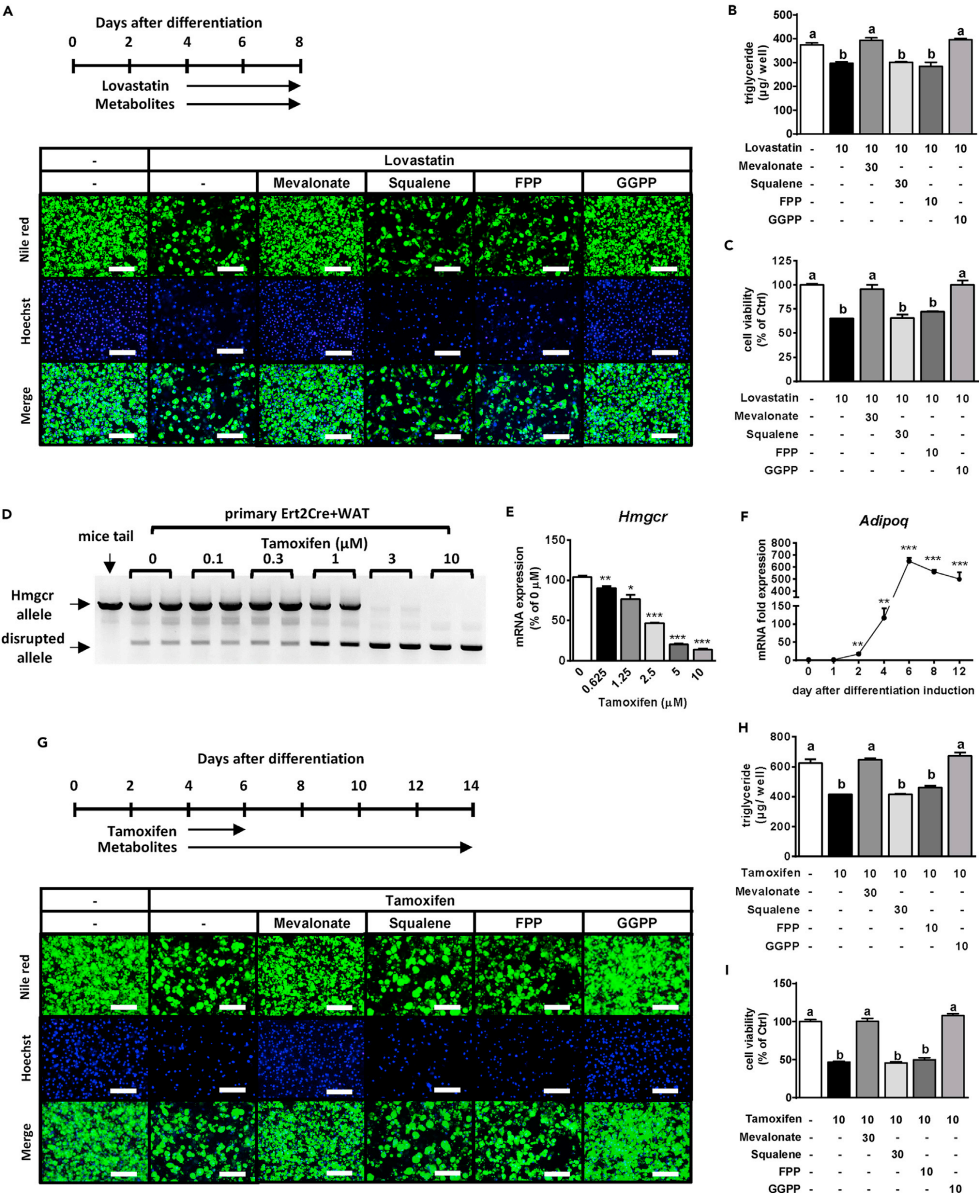
Mevalonate Pathway-Derived Metabolites Are Necessary for Adipocytes (A–C) After the primary WAT cells were differentiated and kept until D4, they were treated with 10 μM Lova in combination with or without MVA metabolites for an additional 4 days (A). The lipids and nuclei in primary WAT cells were stained with Nile red (top panel) and Hoechst 33342 (middle panel), respectively (A). TG accumulation levels (B) and cell viability (C) are also shown. Merge (bottom), the combined images of Nile red and Hoechst. Scale bars, 200 μm. (D and E) After the primary Ert2+ Cre WAT cells were differentiated and kept until D4 to D6, they were treated with Tamo for an additional 48 hr and used for analysis of genomic alleles (D) or *Hmgcr* mRNA levels (E). (F) *Adipoq* mRNA expression during adipocyte differentiation. (G–I) Primary Ert2+ Cre WAT cells were differentiated and kept until D4. Subsequently, the cells were treated with 10 μM Tamo for an additional 48 hr and co-treated with the MVA metabolites until D14 (G). The lipids and nuclei in primary WAT cells were stained with Nile red (top panel) and Hoechst33342 (middle panel), respectively (G). TG accumulation levels (H) and cell viability (I) are also shown. Merge (bottom), the combined images of Nile red and Hoechst. Scale bars, 200 μm. MVA metabolites included 30 μM MVA, 30 μM squalene, 10 μM FPP, and 10 μM GGPP. Bars represent mean ± SE. (n = 6). Bars with different letters represent significant differences (p < 0.05) by one-way ANOVA with a post-hoc Tukey HSD test.

To examine why HMGCR deficiency causes lipodystrophy, we used lovastatin (Lova) to inhibit HMGCR activity in differentiated 3T3-L1 adipocytes. The stained lipid droplets and intracellular TG levels were obviously decreased in Lova-treated 3T3-L1 adipocytes, whereas these phenotypic variations were reversed in the presence of MVA or GGPP but not squalene or FPP (Figures S6A and S6B). Moreover, HMGCR inhibition extensively decreased cell viability, and this effect was prevented by MVA and GGPP (Figure S6C), as evidenced by the number of Hoechst-stained nuclei (Figure S6A). In addition, we isolated primary preadipocytes from the WAT of an HMGCR f/f mouse (primary WAT). Consistent with the other findings, in differentiated primary WAT cells, we found that the Lova-induced reduction in lipid accumulation was reversed by co-treatment with MVA or GGPP but not squalene or FPP (Figures 5A and 5B). Furthermore, the Lova-mediated reduction of adipocyte viability was ameliorated in the presence of MVA or GGPP (Figure 5C). Therefore, Lova-mediated inhibition of the MVA pathway leads to decreased adipocyte viability. Interestingly, MVA and GGPP but not FPP could completely prevent Lova-induced adipocyte death (Figures 5A–5C and S6A–S6C). However, treatment with isopentenyl pyrophosphate (IPP) alone failed to avert Lova-induced reduction of both lipid accumulation and Hoechst-stained nuclei, but successfully rescued these processes when combined with FPP in primary WAT cells (Figure S7). Therefore, both IPP, enzymatically synthesized from MVA, and FPP are required for GGPP synthesis.

To exclude the possibility of adipocyte death caused by cholesterol deficiency, cholesterol was added to Lova-treated primary WAT cells. As shown in Figures S8A and S8B, the Lova-decreased lipid accumulation and Hoechst-stained nuclei in primary WAT cells could not be restored to normal levels by cholesterol treatment. We also fed aKO mice with a high-cholesterol diet (HCD) for 6 weeks. Although the plasma cholesterol level in HCD-fed aKO mice was substantially higher than that in ND-fed aKO mice (Figure S8C), adipose tissue mass and plasma adipokine levels in HCD-fed aKO mice did not recover to normal levels (Figures S8D–S8G). These results clearly suggest that adipocyte death resulting from the inhibition of the MVA pathway was most likely not related to cholesterol insufficiency.

Next, to mimic the HMGCR knockout in adipocytes, we stably induced tamoxifen (Tamo)-activated Cre in primary WAT cells (primary Ert2Cre+ WAT). As shown in Figure 5D, Tamo treatment for 48 hr knocked out the *Hmgcr* gene in a concentration-dependent manner in primary Ert2Cre+ WAT cells. In particular, mRNA expression levels of *Hmgcr* in differentiated primary Ert2Cre+ WAT cells showed concentration-dependent reduction by Tamo treatment (Figure 5E). As we observed that *Adipoq* expression levels in these cells were drastically increased from 4 days after differentiation induction (Figure 5F), we assumed that the knockout program was triggered from D4 to D6. Differentiated primary Ert2Cre+ WAT cells were then treated with 10 μM Tamo and simultaneously with the MVA metabolites, as shown in Figure 5G. The stained lipid droplets and intracellular TG levels were obviously decreased in Tamo-treated adipocytes, whereas these phenotypic variations were reversed in the presence of MVA or GGPP but not squalene or FPP (Figures 5G and 5H). Moreover, Hoechst staining of nuclei and the cell viability assay showed that HMGCR knockout extensively reduced the number of adipocytes and that this effect was prevented by MVA or GGPP treatment (Figures 5G and 5I). These results suggested that the disruption of the MVA synthesis pathway caused adipocyte death, which may be have been due to the lack of GGPP.

### Apoptosis May Be Associated with HMGCR Deficiency-Induced Adipocyte Death

**Figure 6:**
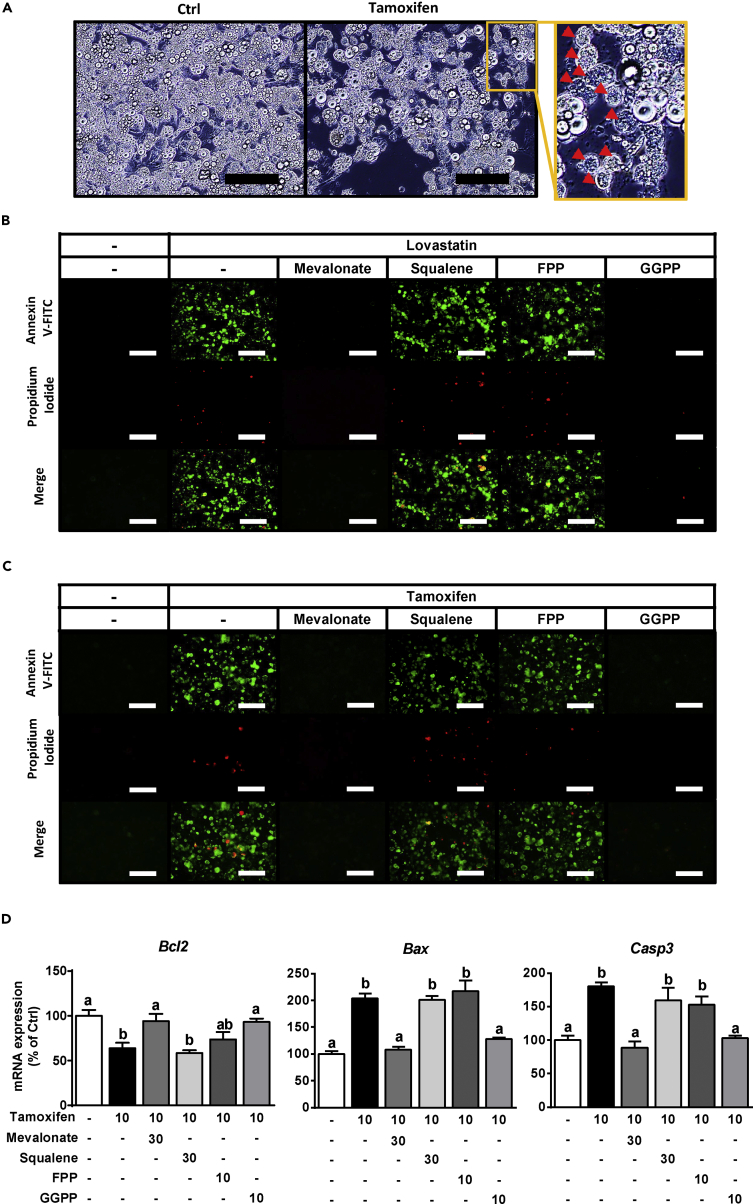
GGPP Is Involved in Adipocyte Survival by Regulating Apoptosis (A) Morphological observation of HMGCR-ablated primary Ert2Cre+ WAT cells. Arrowheads indicate the shrunken adipocytes. Scale bars, 200 μm. (B and C) Annexin V (top panel) and PI (middle panel) staining was performed in primary WAT at D8 (B) and Ert2Cre+ WAT at D14 (C). Merge (bottom panel), the combined images of Annexin V and PI. Scale bars, 200 μm. (D) Apoptosis-related gene expression in primary Ert2Cre+ WAT at D14. Bars represent mean ± SE. (n = 6). Bars with different letters represent significant differences (p < 0.05) by one-way ANOVA with a post-hoc Tukey HSD test.

We observed that smaller cells and cell debris, characteristic of apoptotic cell death, occurred in the disrupted HMGCR adipocytes upon both genetic and pharmacological manipulation (Figures 6A and S9A). Thus, we stained cells with annexin V-fluorescein isothiocyanate (annexin V) and propidium iodide (PI), which indicate early and late apoptosis, respectively. In the Lova-treated 3T3-L1 adipocytes, the numbers of cells stained with annexin V and PI were increased, and this effect was reversed by co-treatment with MVA and GGPP (Figure S9B). Consistent with this finding, Lova-treated primary WAT exhibited almost identical morphological changes (Figure 6B). In addition, Tamo-induced genetic ablation of HMGCR increased the numbers of annexin V- and PI-stained primary Ert2Cre+ WAT cells, and these phenomena were ameliorated by the addition of MVA or GGPP but not squalene or FPP (Figure 6C). Consistent with the results described above, expression of the anti-apoptotic gene B-cell lymphoma 2 (*Bcl-2*) was decreased, whereas that of the apoptotic genes Bcl-2-associated X protein (*Bax*) and caspase 3 (*Casp3*) was increased in HMGCR-knockout adipocytes (Figure 6D). Conversely, these apoptosis-related gene expression changes were dramatically reversed by the administration of MVA or GGPP (Figure 6D). These results indicate that the MVA biosynthesis pathway, especially GGPP, may serve an important role to control adipocyte viability by regulating apoptosis.

### GGPP Plays an Essential Role in Maintaining Adipocyte Survival by Regulating Apoptosis

**Figure 7:**
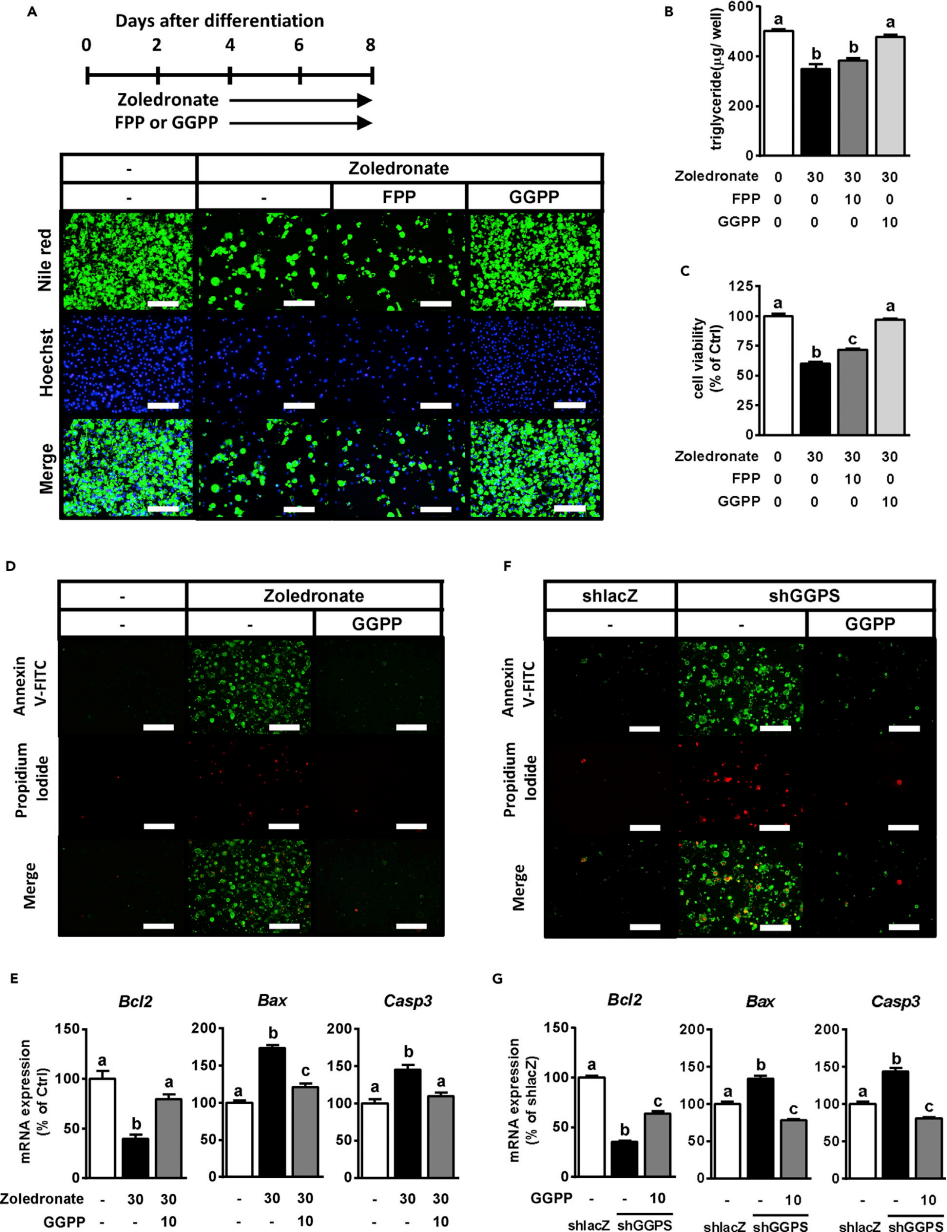
GGPP Is Necessary to Modulate Apoptosis in Adipocytes (A–C) After the primary WAT cells were differentiated and kept until D4, they were treated with 30 μM Zol in combination with or without FPP or GGPP for an additional 4 days (A). The lipids and nuclei in primary WAT cells were stained with Nile red (top panel) and Hoechst 33342 (middle panel), respectively (A). TG accumulation levels (B) and cell viability (C) are also shown. Merge (bottom panel), the combined images of Nile red and Hoechst. Scale bars, 200 μm. (D and E) Annexin V (top panel) and PI (middle panel) staining (D) and measurement of apoptosis-related gene expression (E) were performed in primary WAT at D8. Merge (bottom panel), the combined images of Annexin V and PI. Scale bars, 200 μm. (F and G) Annexin V (top panel) and PI (middle panel) staining (F) and measurement of apoptosis-related gene expression (G) were performed in coxsackievirus-adenovirus receptor-overexpressed 3T3-L1 infected with shlacZ or shGGPS#2 at D8. Merge (bottom panel), the combined images of Annexin V and PI. Scale bars, 200 μm. Bars represent mean ± SE. (n = 6–8). Bars with different letters represent significant differences (p < 0.05) by one-way ANOVA with a post-hoc Tukey HSD test.

To further confirm whether GGPP is the key metabolite necessary for adipocyte survival, we treated primary WAT cells with zoledronate (Zol) to inhibit GGPP synthesis (Guo et al., 2007, No et al., 2012). Zol treatment significantly decreased the lipid accumulation, number of Hoechst-stained nuclei, and cell viability (Figures 7A–7C). However, these Zol-induced effects were completely reversed by GGPP addition and only partially reversed by FPP addition, indicating that GGPP depletion caused adipocyte death (Figures 7A–7C). Moreover, Zol-induced apoptosis, as confirmed by the apoptosis staining assay and apoptosis-related gene expression, was compromised by supplementation of GGPP (Figures 7D and 7E).

Next, GGPP synthase (Ggps) was genetically down-regulated by infection of adenovirus encoding *Ggps*-specific short hairpin RNA (shGGPS) in 3T3-L1 adipocytes overexpressing the coxsackievirus-adenovirus receptor (Figure S10A). Compared with the control group (shlacZ), all types of shGGPS (shGGPS#1, #2, and #3) decreased the expression of *Ggps* and the anti-apoptotic gene *Bcl-2* and enhanced the expression of the apoptotic genes *Bax* and *Casp3*, suggesting that *Ggps* knockdown induced apoptosis in differentiated adipocytes (Figures S10B and S10C). Next, we performed a GGPP add-back experiment to prove that GGPP depletion is the main driver of apoptosis. As shown in Figures S10D–S10F, shGGPS caused adipocyte death, whereas GGPP addition could prevent it. Furthermore, the results of apoptosis staining and relative gene expression showed that GGPP supplementation ameliorated shGGPS-induced adipocyte apoptosis (Figures 7F and 7G). These findings indicate that GGPP is the key metabolite in the MVA pathway, which is indispensable for adipocyte survival, possibly through a mechanism that modulates apoptosis.

## Discussion

The MVA biosynthesis pathway produces not only sterols but also non-sterols termed isoprenoids, such as FPP and GGPP (Casey, 1992, Goldstein and Brown, 1990). Isoprenoids have been shown to play an important role in controlling cell metabolism and viability for both cancer and normal cells, such as fibroblasts, immune-related cells, and neuronal cells (Moutinho et al., 2017, Mullen et al., 2016, Tristano and Fuller, 2006, van der Burgh et al., 2014). Here, we established HMGCR deficiency models *in vivo* and *in vitro* that indicated that the MVA pathway is essential for adipocyte survival, and that suggest that GGPP may contribute in controlling adipocyte death by regulating apoptosis.

In humans, lipodystrophy can broadly be classified into genetic and acquired types (Hegele et al., 2007). Both these lipodystrophies frequently display some metabolic phenomena, such as hepatic disorders, abnormal glucose metabolism, and dyslipidemia (Hegele et al., 2007, Ludtke et al., 2005). Consistent with phenotypic variations resulting from lipodystrophy in humans, in the current study, aKO mice exhibited lipodystrophic phenotypes along with hepatomegaly, severe diabetic symptoms, and abnormal lipid metabolism. Furthermore, these lipodystrophy-related metabolic changes have also been confirmed in different lipodystrophic mouse models, such as the A-ZIP/F1 mouse, which is the typical fatless mouse model (Moitra et al., 1998), or in mice with double knockout for insulin receptor and insulin-like growth factor 1 (Boucher et al., 2016, Softic et al., 2016). Consistent with these lipodystrophic model mice, aKO mice also presented a dramatic decrease in both WAT and BAT mass. However, Guerra et al. (2001) have shown that BAT-atrophic mice exhibit a diabetic phenotype without insulin resistance. These results suggest that at least the diabetic phenotype occurring in aKO mice mainly resulted from atrophy of WAT but not BAT.

The symptoms of lipodystrophic patients or mouse models have largely been reported; however, the phenomena differ based on the respective cause and its mechanisms. In humans, patients with acquired-type lipodystrophies have almost normal weight or are underweight, whereas genetic-type lipodystrophies have different effects on body mass index variation ranging from underweight to overweight according to the mutant gene (Hegele et al., 2007). In comparison, lipodystrophic mice generated by PPARγ disruption exhibit a decrease in body weight gain despite an increase of food intake, energy expenditure, and activity (Jones et al., 2005). The A-ZIP/F1 mouse displayed an obviously enhanced body weight and food intake with no significant variations in energy expenditure and activity (Guo et al., 2012, Moitra et al., 1998). Conversely, in the current study, aKO mice showed increased body weight, food intake, and energy expenditure but decreased locomotor activity. Thus, lipodystrophic phenotypes, such as body weight, food intake, or energy expenditure, possibly vary between cases.

PPARγ mutations represent one genetic type of lipodystrophy (Hegele et al., 2007). Our previous study reported that the MVA-derived metabolite, FPP, acts as a PPARγ ligand (Goto et al., 2011), suggesting that lipodystrophy in aKO mice possibly results from deficiency of a PPARγ ligand. However, Pio treatment failed to reverse the lipodystrophy in aKO mice, whereas it partially improved diabetic phenotypes. In addition, Pio administration significantly increased liver weight and hepatic lipid accumulation but decreased body weight in aKO mice. It has been reported that rosiglitazone treatment improved the glucose metabolism but enhanced liver weight and hepatic lipid accumulation in A-ZIP/F1 mice (Gavrilova et al., 2003). In contrast, in the case of patients with familial partial lipodystrophy, thiazolidinedione (TZD) treatment improved not only diabetic symptoms but also liver disorders such as steatosis and fibrosis (Brown et al., 2016). It is possible that the improved liver function with TZD treatment is due to TZD-induced adipocyte differentiation and proliferation, which helps to reverse the excess hepatic lipid storage associated with insufficient adipose tissue mass (Chao et al., 2004). Combined with these results, our findings suggest that without adequate functional adipose tissue, the liver in aKO mice may constitute the primary and major target organ of TZD action, which is related to systemic glucose and lipid metabolism.

Although local cholesterol deficiency, or that of its metabolites, might contribute to MVA pathway inhibition-induced lipodystrophy in aKO mice, many reports disagree with the cholesterol contribution in lipodystrophy. Severe genetic hypocholesterolemia has been found not to be associated with fat mass loss in humans (Tanoli et al., 2004). Moreover, in the present study, even though the plasma cholesterol level was initially higher in aKO mice and was boosted by HCD, elevated plasma cholesterol seemed to be unable to restore the adipose tissue mass. Consistent results could be found with HMGCR-disrupted adipocytes treated with cholesterol. Although the cause of lipodystrophy in aKO mice seems to weakly relate to cholesterol deficiency, the incapability of *de novo* steroid synthesis was not completely excluded. For instance, in adipocytes, 27-hydroxycholesterol has been identified as one of the major products synthesized *de novo* from cholesterol by Cyp27a1. However, in mice that were deprived of Cyp27a1, no differences in WAT and body weight gain were observed, when compared with wild-type mice (Li et al., 2014). Although further investigation is needed, these results indicate that deficiency of cholesterol, or that of its metabolites, plays a minor causative effect in lipodystrophy in aKO mice.

Notably, both lipodystrophy and obesity have been shown to exhibit several similar metabolic characteristics (Hegele et al., 2007, Ludtke et al., 2005), suggesting that adipose tissue plays an important role in metabolic homeostasis. Previous reports demonstrated that implantation of adipose tissue reversed the diabetic symptoms in A-ZIP/F1 mice (Gavrilova et al., 2000). Similarly, the present results showed that adipose tissue implantation led to dramatic recovery in all metabolic disorders in aKO mice. These phenotypic effects may have been caused by several mechanisms. First, transplanted fat grafts functionally secrete adipokines, such as adiponectin and leptin, which may contribute to the metabolic improvement. Yamauchi et al. (2001) reported that lipodystrophy-induced insulin resistance was completely reversed by combined treatment of adiponectin and leptin. In addition, it has been found that transplantation of leptin-deficient adipose tissue only minimally improved lipodystrophy-related metabolic abnormalities in A-ZIP/F1 mice (Colombo et al., 2002). Colombo et al. (2002) also showed that the contribution of fat transplantation to plasma adiponectin is very low and that leptin treatment improved metabolism without any changes in adiponectin levels. Consistent with this finding, leptin treatment and overexpression of leptin also yielded an improvement in glucose metabolism and hepatic lipid accumulation in Srebp-1c Tg and A-ZIP/F1 mice, respectively (Ebihara et al., 2001, Shimomura et al., 1999). These observations suggest that leptin is possibly a major adipokine required for the regulation of glucose and lipid metabolism when adipose tissue is lacking. Second, implanted fat grafts allow the storage of lipids such as FFA, cholesterol, and TG. Gavrilova et al. (2000) reported that at 13 weeks after transplantation in A-ZIP/F1 mice, the total weight of transplanted fat increased by approximately 1.4-fold, and that the improvement of metabolic disorders was dose dependent with transplanted fat mass. In addition, low-mass fat implantation also ameliorated the disruption of glucose and lipid metabolism without any increase of leptin (Gavrilova et al., 2000). Furthermore, TZD improved hepatic and diabetic disorders in patients with lipoatrophy concomitant with enhanced adipose tissue mass (Brown et al., 2016). In the present study, both transplanted fat pad mass and adipocyte size were enhanced, indicating that the recovered capacity for storage of lipids in adipose tissue may contribute to the improvement of metabolic disorders caused by lipodystrophy. Moreover, fat transplantation also enhanced the increase of plasma leptin in aKO mice by 14-fold, despite an overall recovery to only 35% of the level of Ctrl mice. However, whether this enhanced plasma leptin level is sufficient to repress the abnormal metabolism in aKO mice remains unclear and requires further investigation.

The MVA pathway has been implicated in multiple aspects of the cell cycle including cell differentiation, survival, and growth (Bonetti et al., 2003, Demierre et al., 2005). For example, it has been reported that the MVA pathway is essential for regulation of the proliferation and growth of vascular smooth muscle cells (Bonetti et al., 2003). Several cancers, such as colorectal cancer and melanoma, were inhibited by administration of HMGCR inhibitors (Demierre et al., 2005). These reports suggested that MVA pathway inhibition is possibly linked to programmed cell death, i.e., apoptosis (Bonetti et al., 2003, Demierre et al., 2005, Wong et al., 2002). Similarly, in adipocytes, we found that a disrupted MVA pathway caused cell death that at least partially involved isoprenoid-regulated apoptosis. The possible involvement of coenzyme Q10 (CoQ) deficiency or impaired GGPP-related prenylation has been reported in several cell lines, such as myocytes, hepatocytes, or tumor cells, as the underlying mechanism of HMGCR inhibition-induced apoptosis (Alizadeh et al., 2017, Auer et al., 2016, Demierre et al., 2005, Tavintharan et al., 2007). However, CoQ supplementation did not prevent Lova-induced adipocyte death, and treatment of GGTI2133 and Y27632, inhibitors of geranylgeranyl transferase and Rho-associated protein kinase, respectively, did not alter the outcome of adipocyte death (data not shown), indicating that the relationship between GGPP and apoptosis is an unexpected mechanism. Although detailed information about the mechanism remains unclear, we urge persistent users of statins to take the findings of this study into consideration. Interestingly, supplementation with FPP failed to prevent HMGCR inhibition-induced cell death, although GGPP is enzymatically synthesized from FPP. This may have occurred because IPP deficiency, resulting from HMGCR impairment, could not convert FPP to GGPP (Demierre et al., 2005, Park et al., 2014), which was also confirmed in the present report. In addition, Pajvani et al. (2005) demonstrated that adipose-targeted apoptosis causes lipodystrophy. These results may underlie the strong relationship between lipodystrophy and apoptosis in aKO mice.

Obesity, a complex multifaceted disease based on an increased WAT mass, is associated with an enhanced risk for other metabolic diseases such as diabetes mellitus (Kusminski et al., 2016). Notably, adipocyte turnover is extremely low in humans, such that the adipocytes replace themselves in about 10 years, whereas most other cell types only require from several days to a few months (Shamir et al., 2016, Spalding et al., 2008). It appears that adipocyte death is rarely triggered, although the detailed mechanisms remain unclear. Previous reports have demonstrated that intracellular GGPP levels and Ggps expression levels increase during adipocyte differentiation. Moreover, *Ggps* gene expression levels were enhanced in WAT in both HFD-induced and ob/ob obese mouse models, as well as in diabetic db/db mice (Yeh et al., 2016, Yu et al., 2011), suggesting that GGPP may play a major role in adipocyte function. In the present study, we show that HMGCR deficiency contributed to lipodystrophy, and that this may be associated with GGPP-related regulation of apoptosis. Here, we hypothesize that GGPP is a key MVA-derived metabolite involved in maintaining adipocyte survival. Upon adipocyte expansion and maturation, increased GGPP levels may protect adipocytes from apoptosis. This hypothesis potentially suggests that adipocyte turnover may be regulated through modulation of the MVA pathway. However, further investigation is needed to clarify this hypothesis.

Overall, our results demonstrate that adipose-specific disruption of the MVA pathway causes lipodystrophy and results in several metabolic variations such as abnormal glucose and lipid metabolism, as well as hepatic disorders. Here, we propose one possible mechanism wherein GGPP deficiency in adipocytes underlies these phenotypes. Moreover, the lipodystrophy mediated by MVA pathway inhibition possibly resulted from GGPP deficiency-induced adipocyte apoptosis. Our hypothesis provides insight regarding the potential mechanisms to control adipocyte turnover through regulation of the MVA pathway, as a potential therapeutic strategy for obesity. Furthermore, these findings might provide useful information for the clinical application of statin.

### Limitations of the Study

We showed that GGPP deficiency induced apoptosis in adipocytes, suggesting that the MVA pathway is critical for adipocyte survival. However, owing to technical limitations, we could not quantify GGPP in adipocytes and adipose tissue. Therefore, we do not know the exact amount of GGPP required to ensure adipocyte survival. A main concern is regarding why blood circulation could not provide sufficient MVA or GGPP to compensate the HMGCR deficiency-induced lipodystrophy. Furthermore, we do not know whether the role of inhibited HMGCR in human adipocyte is consistent with that shown in the present study, because we used a mouse and mouse-derived cell models. Although we cannot provide any information about these issues, we aim to address such important issues in future studies to better understand the physiological function of GGPP.

## Methods

All methods can be found in the accompanying Transparent Methods supplemental file.

## Supplemental Information

Document S1. Transparent Methods, Figures S1–S10, and Tables S1 and S2

## References

[bib1] Alizadeh J., Zeki A.A., Mirzaei N., Tewary S., Rezaei Moghadam A., Glogowska A., Nagakannan P., Eftekharpour E., Wiechec E., Gordon J.W. (2017). Mevalonate cascade inhibition by simvastatin induces the intrinsic apoptosis pathway via depletion of isoprenoids in tumor cells. Sci. Rep..

[bib2] Auer J., Sinzinger H., Franklin B., Berent R. (2016). Muscle- and skeletal-related side-effects of statins: tip of the iceberg?. Eur. J. Prev. Cardiol..

[bib3] Bang C.N., Okin P.M. (2014). Statin treatment, new-onset diabetes, and other adverse effects: a systematic review. Curr. Cardiol. Rep..

[bib4] Bays H.E., Toth P.P., Kris-Etherton P.M., Abate N., Aronne L.J., Brown W.V., Gonzalez-Campoy J.M., Jones S.R., Kumar R., La Forge R. (2013). Obesity, adiposity, and dyslipidemia: a consensus statement from the National Lipid Association. J. Clin. Lipidol..

[bib5] Bonetti P.O., Lerman L.O., Napoli C., Lerman A. (2003). Statin effects beyond lipid lowering–are they clinically relevant?. Eur. Heart J..

[bib6] Boucher J., Softic S., El Ouaamari A., Krumpoch M.T., Kleinridders A., Kulkarni R.N., O'Neill B.T., Kahn C.R. (2016). Differential roles of insulin and IGF-1 receptors in adipose tissue development and function. Diabetes.

[bib7] Brault M., Ray J., Gomez Y.H., Mantzoros C.S., Daskalopoulou S.S. (2014). Statin treatment and new-onset diabetes: a review of proposed mechanisms. Metabolism.

[bib8] Brown R.J., Araujo-Vilar D., Cheung P.T., Dunger D., Garg A., Jack M., Mungai L., Oral E.A., Patni N., Rother K.I. (2016). The diagnosis and management of lipodystrophy syndromes: a multi-society practice guideline. J. Clin. Endocrinol. Metab..

[bib9] Casey P.J. (1992). Biochemistry of protein prenylation. J. Lipid Res..

[bib10] Chao P.J., Tsai J.C., Chang D.M., Shin S.J., Lee Y.J. (2004). A case of acquired generalized lipodystrophy with cerebellar degeneration and type 2 diabetes mellitus. The review of diabetic studies. Rev. Diabet. Stud..

[bib11] Colombo C., Cutson J.J., Yamauchi T., Vinson C., Kadowaki T., Gavrilova O., Reitman M.L. (2002). Transplantation of adipose tissue lacking leptin is unable to reverse the metabolic abnormalities associated with lipoatrophy. Diabetes.

[bib12] Demierre M.F., Higgins P.D., Gruber S.B., Hawk E., Lippman S.M. (2005). Statins and cancer prevention. Nat. Rev. Cancer.

[bib13] Ebihara K., Ogawa Y., Masuzaki H., Shintani M., Miyanaga F., Aizawa-Abe M., Hayashi T., Hosoda K., Inoue G., Yoshimasa Y. (2001). Transgenic overexpression of leptin rescues insulin resistance and diabetes in a mouse model of lipoatrophic diabetes. Diabetes.

[bib14] Eguchi J., Wang X., Yu S., Kershaw E.E., Chiu P.C., Dushay J., Estall J.L., Klein U., Maratos-Flier E., Rosen E.D. (2011). Transcriptional control of adipose lipid handling by IRF4. Cell Metab..

[bib15] Fasshauer M., Bluher M. (2015). Adipokines in health and disease. Trends Pharmacol. Sci..

[bib16] Gavrilova O., Haluzik M., Matsusue K., Cutson J.J., Johnson L., Dietz K.R., Nicol C.J., Vinson C., Gonzalez F.J., Reitman M.L. (2003). Liver peroxisome proliferator-activated receptor gamma contributes to hepatic steatosis, triglyceride clearance, and regulation of body fat mass. J. Biol. Chem..

[bib17] Gavrilova O., Marcus-Samuels B., Graham D., Kim J.K., Shulman G.I., Castle A.L., Vinson C., Eckhaus M., Reitman M.L. (2000). Surgical implantation of adipose tissue reverses diabetes in lipoatrophic mice. J. Clin. Invest..

[bib18] Goldstein J.L., Brown M.S. (1990). Regulation of the mevalonate pathway. Nature.

[bib19] Goto T., Nagai H., Egawa K., Kim Y.I., Kato S., Taimatsu A., Sakamoto T., Ebisu S., Hohsaka T., Miyagawa H. (2011). Farnesyl pyrophosphate regulates adipocyte functions as an endogenous PPARgamma agonist. Biochem. J..

[bib20] Guerra C., Navarro P., Valverde A.M., Arribas M., Bruning J., Kozak L.P., Kahn C.R., Benito M. (2001). Brown adipose tissue-specific insulin receptor knockout shows diabetic phenotype without insulin resistance. J. Clin. Invest..

[bib21] Guo R.T., Cao R., Liang P.H., Ko T.P., Chang T.H., Hudock M.P., Jeng W.Y., Chen C.K., Zhang Y., Song Y. (2007). Bisphosphonates target multiple sites in both cis- and trans-prenyltransferases. Proc. Natl. Acad. Sci. U S A.

[bib22] Guo T., Bond N.D., Jou W., Gavrilova O., Portas J., McPherron A.C. (2012). Myostatin inhibition prevents diabetes and hyperphagia in a mouse model of lipodystrophy. Diabetes.

[bib23] Hegele R.A., Joy T.R., Al-Attar S.A., Rutt B.K. (2007). Thematic review series: adipocyte Biology. Lipodystrophies: windows on adipose biology and metabolism. J. Lipid Res..

[bib24] Jones J.R., Barrick C., Kim K.A., Lindner J., Blondeau B., Fujimoto Y., Shiota M., Kesterson R.A., Kahn B.B., Magnuson M.A. (2005). Deletion of PPARgamma in adipose tissues of mice protects against high fat diet-induced obesity and insulin resistance. Proc. Natl. Acad. Sci. U S A.

[bib25] Kamal S., Khan M.A., Seth A., Cholankeril G., Gupta D., Singh U., Kamal F., Howden C.W., Stave C., Nair S. (2017). Beneficial effects of statins on the rates of hepatic fibrosis, hepatic decompensation, and mortality in chronic liver disease: a systematic review and meta-analysis. Am. J. Gastroenterol..

[bib26] Krause B.R., Hartman A.D. (1984). Adipose tissue and cholesterol metabolism. J. Lipid Res..

[bib27] Kusminski C.M., Bickel P.E., Scherer P.E. (2016). Targeting adipose tissue in the treatment of obesity-associated diabetes. Nat. Rev. Drug Discov..

[bib28] Lackey D.E., Olefsky J.M. (2016). Regulation of metabolism by the innate immune system. Nat. Rev. Endocrinol..

[bib29] Li J., Daly E., Campioli E., Wabitsch M., Papadopoulos V. (2014). De novo synthesis of steroids and oxysterols in adipocytes. J. Biol. Chem..

[bib30] Ludtke A., Genschel J., Brabant G., Bauditz J., Taupitz M., Koch M., Wermke W., Worman H.J., Schmidt H.H. (2005). Hepatic steatosis in Dunnigan-type familial partial lipodystrophy. Am. J. Gastroenterol..

[bib31] Moitra J., Mason M.M., Olive M., Krylov D., Gavrilova O., Marcus-Samuels B., Feigenbaum L., Lee E., Aoyama T., Eckhaus M. (1998). Life without white fat: a transgenic mouse. Genes Dev..

[bib32] Moutinho M., Nunes M.J., Rodrigues E. (2017). The mevalonate pathway in neurons: it's not just about cholesterol. Exp. Cell Res..

[bib33] Mullen P.J., Yu R., Longo J., Archer M.C., Penn L.Z. (2016). The interplay between cell signalling and the mevalonate pathway in cancer. Nat. Rev. Cancer.

[bib34] Nagashima S., Yagyu H., Ohashi K., Tazoe F., Takahashi M., Ohshiro T., Bayasgalan T., Okada K., Sekiya M., Osuga J. (2012). Liver-specific deletion of 3-hydroxy-3-methylglutaryl coenzyme A reductase causes hepatic steatosis and death. Arterioscler. Thromb. Vasc. Biol..

[bib35] No J.H., de Macedo Dossin F., Zhang Y., Liu Y.L., Zhu W., Feng X., Yoo J.A., Lee E., Wang K., Hui R. (2012). Lipophilic analogs of zoledronate and risedronate inhibit Plasmodium geranylgeranyl diphosphate synthase (GGPPS) and exhibit potent antimalarial activity. Proc. Natl. Acad. Sci. U S A.

[bib36] Osaki Y., Nakagawa Y., Miyahara S., Iwasaki H., Ishii A., Matsuzaka T., Kobayashi K., Yatoh S., Takahashi A., Yahagi N. (2015). Skeletal muscle-specific HMG-CoA reductase knockout mice exhibit rhabdomyolysis: a model for statin-induced myopathy. Biochem. Biophys. Res. Commun..

[bib37] Pajvani U.B., Trujillo M.E., Combs T.P., Iyengar P., Jelicks L., Roth K.A., Kitsis R.N., Scherer P.E. (2005). Fat apoptosis through targeted activation of caspase 8: a new mouse model of inducible and reversible lipoatrophy. Nat. Med..

[bib38] Park J., Matralis A.N., Berghuis A.M., Tsantrizos Y.S. (2014). Human isoprenoid synthase enzymes as therapeutic targets. Front. Chem..

[bib39] Shamir M., Bar-On Y., Phillips R., Milo R. (2016). SnapShot: timescales in cell biology. Cell.

[bib40] Shimomura I., Hammer R.E., Ikemoto S., Brown M.S., Goldstein J.L. (1999). Leptin reverses insulin resistance and diabetes mellitus in mice with congenital lipodystrophy. Nature.

[bib41] Softic S., Boucher J., Solheim M.H., Fujisaka S., Haering M.F., Homan E.P., Winnay J., Perez-Atayde A.R., Kahn C.R. (2016). Lipodystrophy due to adipose tissue-specific insulin receptor knockout results in progressive NAFLD. Diabetes.

[bib42] Spalding K.L., Arner E., Westermark P.O., Bernard S., Buchholz B.A., Bergmann O., Blomqvist L., Hoffstedt J., Naslund E., Britton T. (2008). Dynamics of fat cell turnover in humans. Nature.

[bib43] Tanoli T., Yue P., Yablonskiy D., Schonfeld G. (2004). Fatty liver in familial hypobetalipoproteinemia: roles of the APOB defects, intra-abdominal adipose tissue, and insulin sensitivity. J. Lipid Res..

[bib44] Tavintharan S., Ong C.N., Jeyaseelan K., Sivakumar M., Lim S.C., Sum C.F. (2007). Reduced mitochondrial coenzyme Q10 levels in HepG2 cells treated with high-dose simvastatin: a possible role in statin-induced hepatotoxicity?. Toxicol. Appl. Pharmacol..

[bib45] Thompson P.D., Clarkson P., Karas R.H. (2003). Statin-associated myopathy. JAMA.

[bib46] Tristano A.G., Fuller K. (2006). Immunomodulatory effects of statins and autoimmune rheumatic diseases: novel intracellular mechanism involved. Int. Immunopharmacol..

[bib47] van der Burgh R., Nijhuis L., Pervolaraki K., Compeer E.B., Jongeneel L.H., van Gijn M., Coffer P.J., Murphy M.P., Mastroberardino P.G., Frenkel J. (2014). Defects in mitochondrial clearance predispose human monocytes to interleukin-1beta hypersecretion. J. Biol. Chem..

[bib48] Wadhera R.K., Steen D.L., Khan I., Giugliano R.P., Foody J.M. (2016). A review of low-density lipoprotein cholesterol, treatment strategies, and its impact on cardiovascular disease morbidity and mortality. J. Clin. Lipidol..

[bib49] Wong W.W., Dimitroulakos J., Minden M.D., Penn L.Z. (2002). HMG-CoA reductase inhibitors and the malignant cell: the statin family of drugs as triggers of tumor-specific apoptosis. Leukemia.

[bib50] Yamauchi T., Kamon J., Waki H., Terauchi Y., Kubota N., Hara K., Mori Y., Ide T., Murakami K., Tsuboyama-Kasaoka N. (2001). The fat-derived hormone adiponectin reverses insulin resistance associated with both lipoatrophy and obesity. Nat. Med..

[bib51] Yeh Y.S., Goto T., Takahashi N., Egawa K., Takahashi H., Jheng H.F., Kim Y.I., Kawada T. (2016). Geranylgeranyl pyrophosphate performs as an endogenous regulator of adipocyte function via suppressing the LXR pathway. Biochem. Biophys. Res. Commun..

[bib52] Yu X., Shen N., Zhang M.L., Pan F.Y., Wang C., Jia W.P., Liu C., Gao Q., Gao X., Xue B. (2011). Egr-1 decreases adipocyte insulin sensitivity by tilting PI3K/Akt and MAPK signal balance in mice. EMBO J..

[bib53] Zaharan N.L., Williams D., Bennett K. (2013). Statins and risk of treated incident diabetes in a primary care population. Br. J. Clin. Pharmacol..

