# Supplementary material for: The Mevalonate Pathway Is Indispensable for Adipocyte Survival
Source: iScience. 2018 Oct 21;9:175–91. doi: 10.1016/j.isci.2018.10.019 (PMC6223239; doi:10.1016/j.isci.2018.10.019)
Supplement: Document S1. Transparent Methods, Figures S1–S10, and Tables S1 and S2 [file mmc1.pdf]

ISCI, Volume *9*

Supplemental Information

The Mevalonate Pathway Is Indispensable for Adipocyte Survival

Yu-Sheng Yeh, Huei-Fen Jheng, Mari Iwase, Minji Kim, Shinsuke Mohri, Jungin Kwon, Satoko Kawarasaki, Yongjia Li, Haruya Takahashi, Takeshi Ara, Wataru Nomura, Teruo Kawada, and Tsuyoshi Goto

Supplemental figures and legends

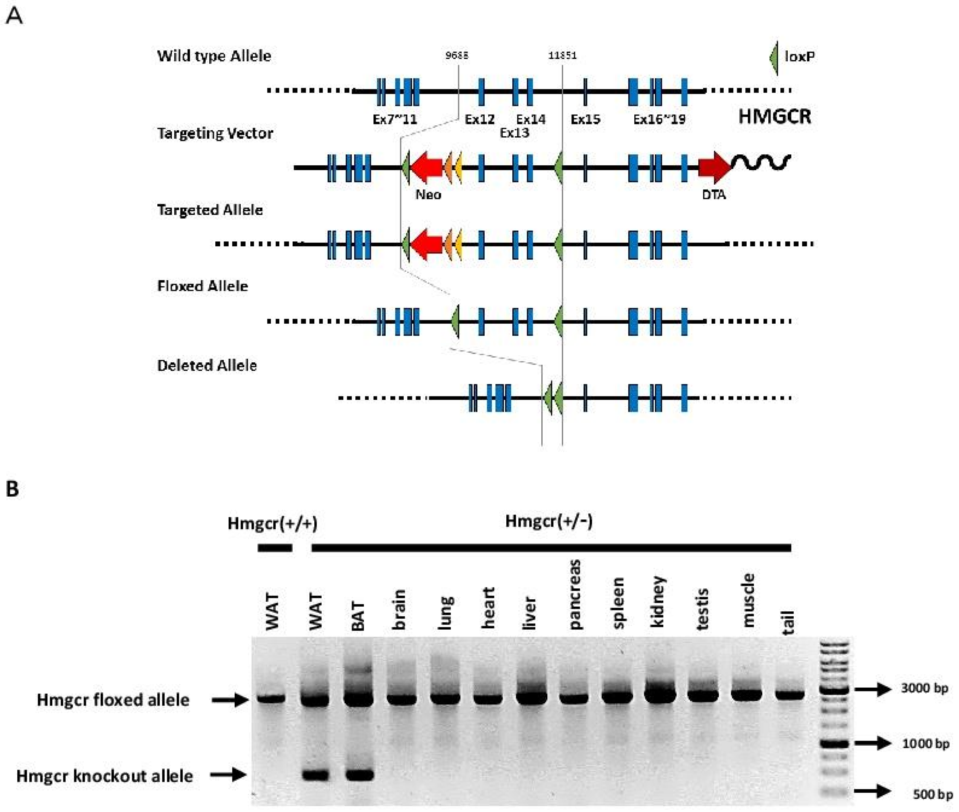

Figure S1. Specific Disruption of the *Hmgcr*, Related to Figure 1 and 2

(A) Strategy for conditional deletion of *Hmgcr*

(B) DNA was amplified by PCR to check the *Hmgcr* alleles. The floxed and knockout alleles are 2757 and 519 bp, respectively.

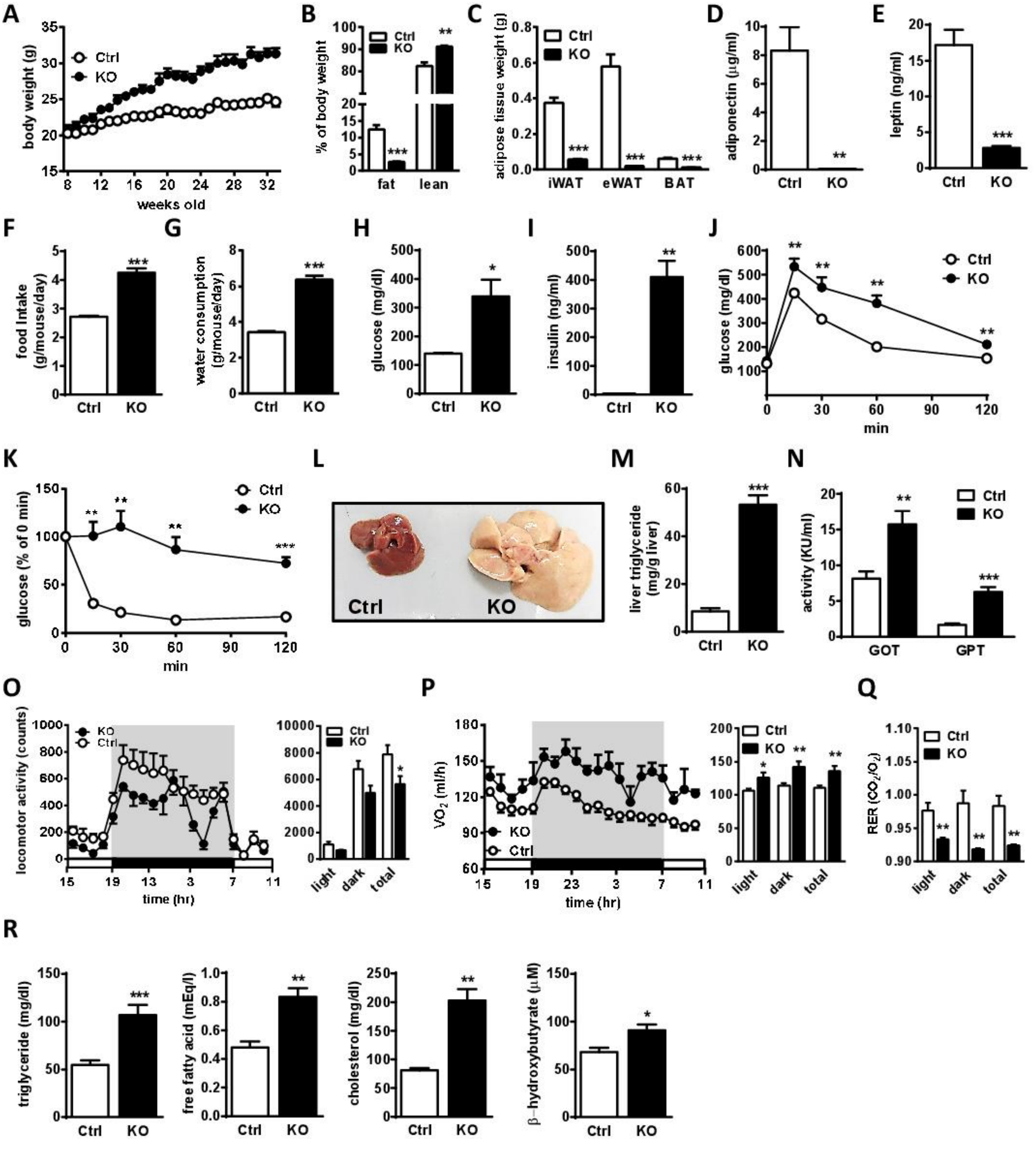

Figure S2. Lipodystrophy in Female aKO Mice and its Phenotypes, Related to Figure 1 and 2

(A) Body weight change of female control (Ctrl) and aKO mice.

(B) Relative fat and lean mass levels of 15-week-old female Ctrl and aKO mice.

(C) Adipose tissue weight of female Ctrl and aKO mice at 32 weeks of age.

(D and E) Plasma adiponectin (D) and leptin (E) levels in female Ctrl and aKO mice quantified at 32 weeks of age.

(F and G) Food intake (F) and water consumption (G) of female Ctrl and aKO mice as measured from 14 to 32 weeks of age.

(H and I) Plasma glucose (H) and insulin (I) levels as analyzed in the 32-week-old female Ctrl and aKO mice.

(J) GTT in female Ctrl and aKO mice as performed at 16 weeks of age.

(K) ITT in female Ctrl and aKO mice as performed at 18 weeks of age.

(L) Gross morphology of the liver in female Ctrl and aKO mice at 32 weeks of age.

(M) Hepatic lipid accumulation levels in 32-week-old female Ctrl and aKO mice.

(N) Plasma GOT and GPT activity in 32-week-old Ctrl and aKO mice.

(O-Q) Locomotor activity (O), oxygen consumption (P), and RER (Q) as detected from 20 to 25 weeks of age in female Ctrl and aKO mice.

(R) Plasma TG, FFA, cholesterol, and β-HB levels in 32-week-old female Ctrl and aKO mice.

All mice were fed ND. Bars represent the mean ± SE. (n = 4–8). Significant differences were determined by Student's *t* test compared with Ctrl: *$p < 0.05$, **$p < 0.01$, ***$p < 0.001$.

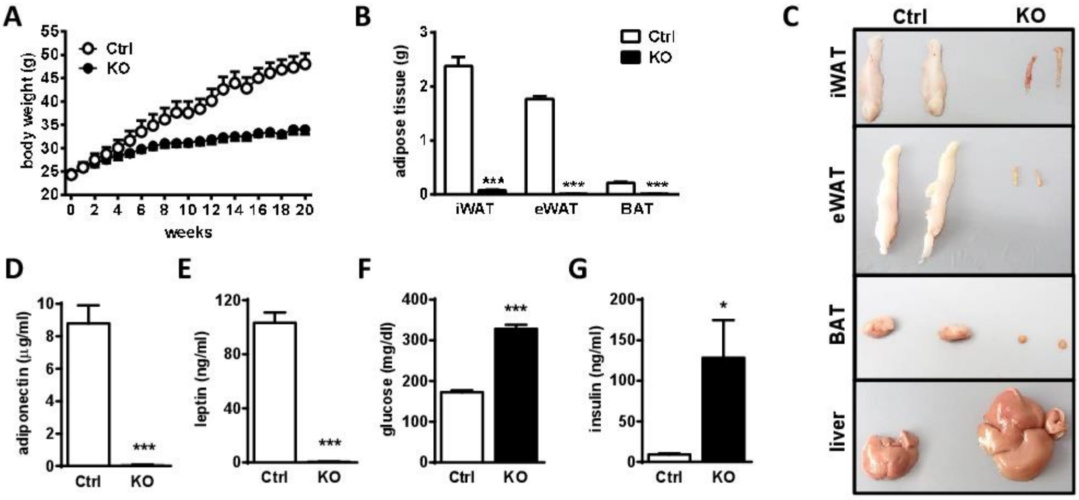

Figure S3. Effect of HFD on Lipodystrophic aKO Mice, Related to Figure 1 and 2

(A) Body weight change of control (Ctrl) and aKO mice from 8 weeks of age.

(B) Adipose tissue weights of Ctrl and aKO mice.

(C) Gross morphology of iWAT, eWAT, BAT, and the liver (from top to bottom) of Ctrl and aKO mice.

(D and E) Plasma adiponectin (D) and leptin (E) levels in Ctrl and aKO mice.

(F and G) Plasma glucose (F) and insulin (G) levels as analyzed in the Ctrl and aKO mice.

These results were obtained after administration of HFD for 20 weeks in 8-week-old Ctrl and aKO mice. Bars represent the mean ± SE. (n = 6). Significant differences were determined by Student's *t* test compared with Ctrl: $^{*}p < 0.05$, $^{***}p < 0.001$.

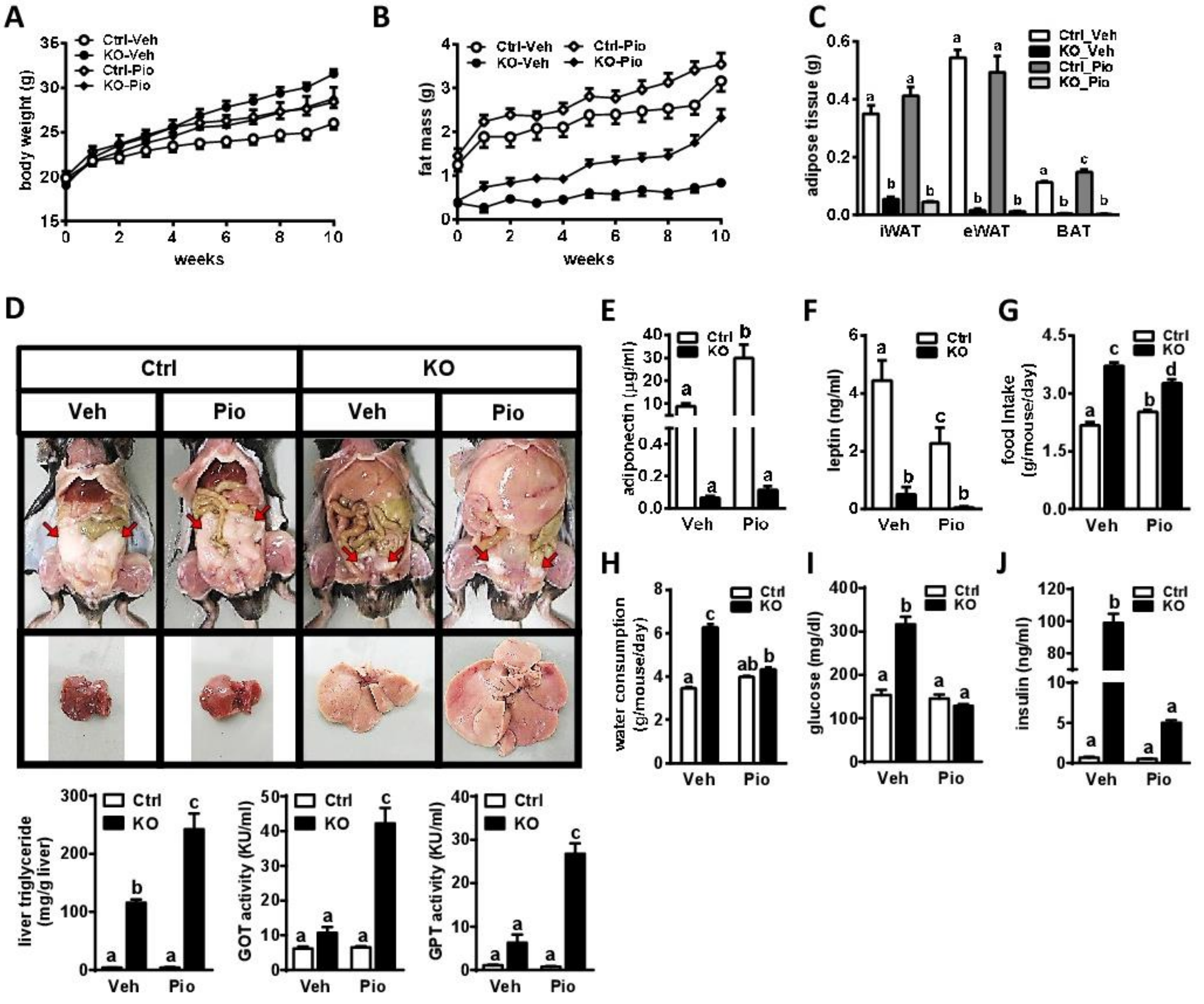

Figure S4. Pio Treatment Partially Improves Diabetic Phenotypes in aKO Mice Without Increased Adipose Tissue Mass, Related to Figure 1 and 2

(A and B) Body weight (A) and relative fat mass (B) changes of control (Ctrl) and aKO mice during Pio treatment.

(C) Adipose tissue weights of Ctrl and aKO mice after 10-week Pio treatment.

(D) Gross morphology of eWAT (top panel) and the liver (bottom panel). Hepatic TG accumulation levels and plasma GOT and GPT activity were measured.

(E and F) Plasma adiponectin (E) and leptin (F) levels of Ctrl and aKO mice after 10-week Pio treatment.

(G and H) Food intake (G) and water consumption (H) of Ctrl and aKO mice as measured from 5 to 10 weeks after Pio treatment.

(I and J) Plasma glucose (I) and insulin (J) levels of Ctrl and aKO mice after 10-week Pio treatment.

Bars represent the mean ± SE. ($n = 6–7$). Bars with different letters represent significant differences ($p < 0.05$) by one-way ANOVA with a post-hoc Tukey HSD test. Veh, vehicle.

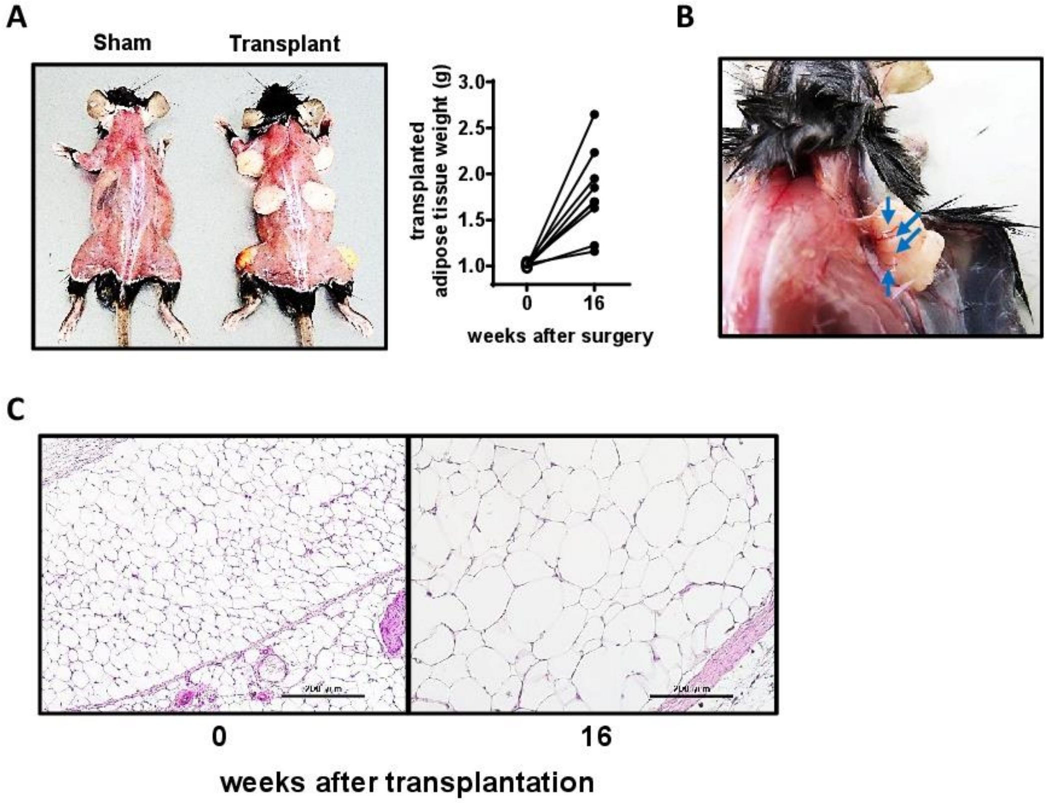

Figure S5. Successful WAT Transplantation as Confirmed in aKO Mice, Related to Figure 3 and 4

(A) Gross morphology of the back (left) and weight change (right) of transplanted WAT at 16 weeks after surgery.

(B) At 2 weeks after fat implantation, blood vessels were confirmed in fat grafts. Arrows indicate the blood vessels.

(C) Sections from adipose tissue before and at 16 weeks after transplantation as stained with H&E. Scale bars represent 200 μm.

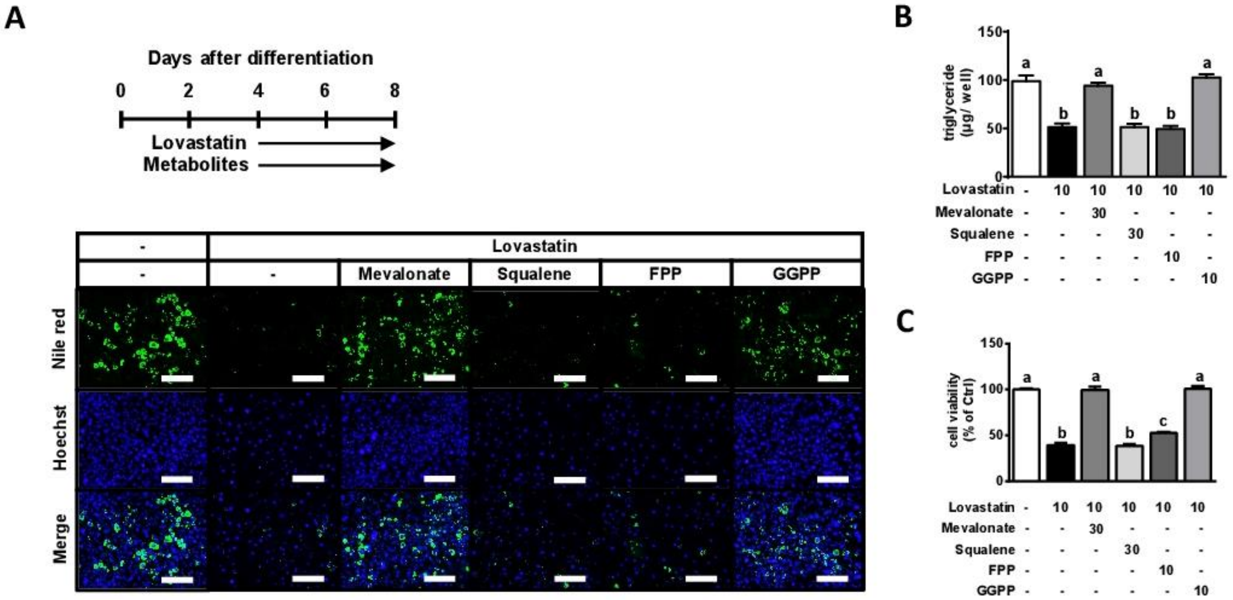

Figure S6. Effect of Lova-Inhibited HMGCR on Differentiated 3T3-L1 Adipocytes, Related to Figure 5

(A-C) After 3T3-L1 cells were differentiated and kept until D4, they were treated with 10 μM Lova in combination with or without MVA metabolites for an additional 4 days (A). The lipids and nuclei were stained with Nile red (top panel) and Hoechst 33324 (middle panel) (A). Lipid accumulation levels (B) and cell viability (C). Scale bars represent 200 μm. MVA metabolites included 30 μM MVA, 30 μM squalene, 10 μM FPP, and 10 μM GGPP. Bars represent the mean ± SE. (n = 6). Bars with different letters represent significant differences ($p < 0.05$) by one-way ANOVA with a post-hoc Tukey HSD test.

A

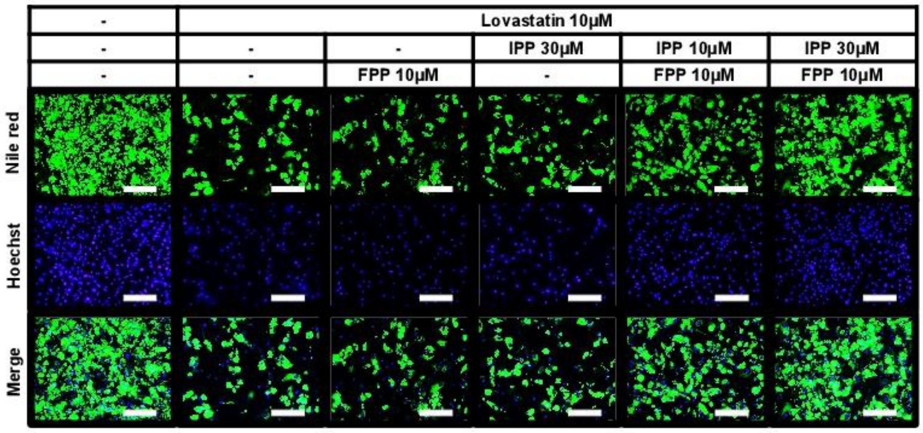

B

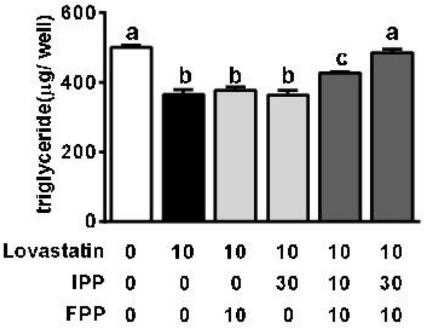

Figure S7. Combination of FPP and IPP Can Prevent Lova-induced Adipocyte death, Related to Figure 5

(A-C) After primary WAT cells were differentiated and kept until D4, they were treated with 10 μM Lova in combination with or without MVA metabolites for an additional 4 days (A). The lipids and nuclei were stained with Nile red (top panel) and Hoechst 33324 (middle panel) (A). Lipid accumulation levels (B) Scale bars represent 200 μm.

MVA metabolites included 10 or 30 μM IPP, 10 μM FPP, and 10 μM GGPP. Bars represent the mean ± SE. (n = 3-4). Bars with different letters represent significant differences ($p < 0.05$) by one-way ANOVA with a post-hoc Tukey HSD test.

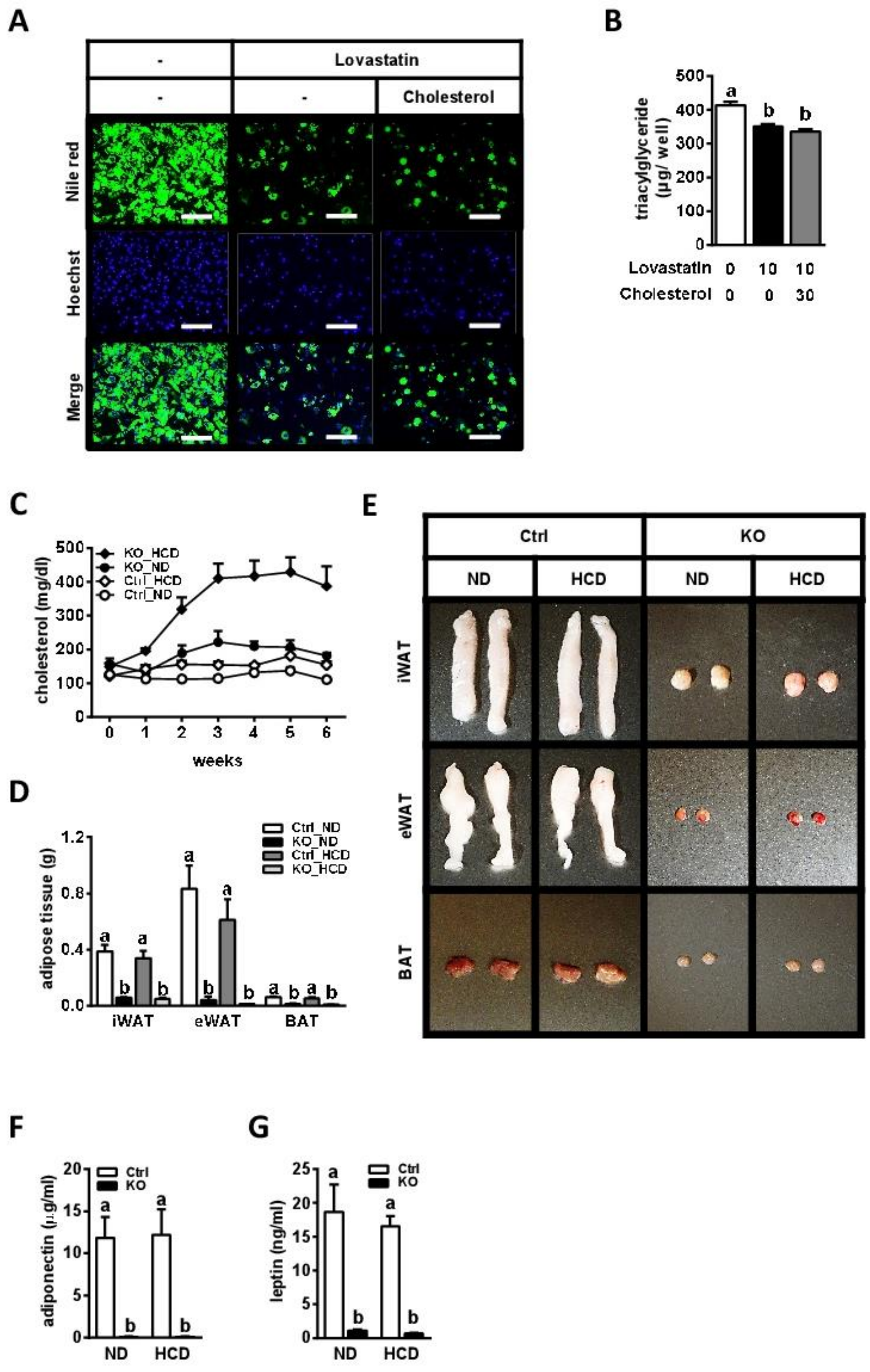

Figure S8. Cholesterol Treatment Fails to Prevent the Adipocyte Death Caused by MVA Pathway Inhibition, Related to Figure 5

(A and B) After primary WAT cells were differentiated and kept until D4, they were treated with 10 μM Lova in combination with or without 30 μM cholesterol for an additional 4 days. The lipids and nuclei were stained with Nile red (top panel) and Hoechst 33324 (middle panel) (A). Lipid accumulation levels (B). Scale bars represent 200 μm.

Bars represent the mean ± SE. (n = 6). Bars with different letters represent significant differences ($p < 0.05$) by one-way ANOVA with a post-hoc Tukey HSD test.

(C) Plasma cholesterol levels of Ctrl and aKO mice during feeding HCD.

(D) Adipose tissue weights of Ctrl and aKO mice after 6-week HCD treatment.

(E) Gross morphology of iWAT (top panel) eWAT (middle panel) and BAT (bottom panel) after 6-week HCD treatment.

(F and G) Plasma adiponectin (F) and leptin (G) levels of Ctrl and aKO mice after 6-week HCD treatment.

Bars represent the mean ± SE. (n = 4). Bars with different letters represent significant differences ($p < 0.05$) by one-way ANOVA with a post-hoc Tukey HSD test.

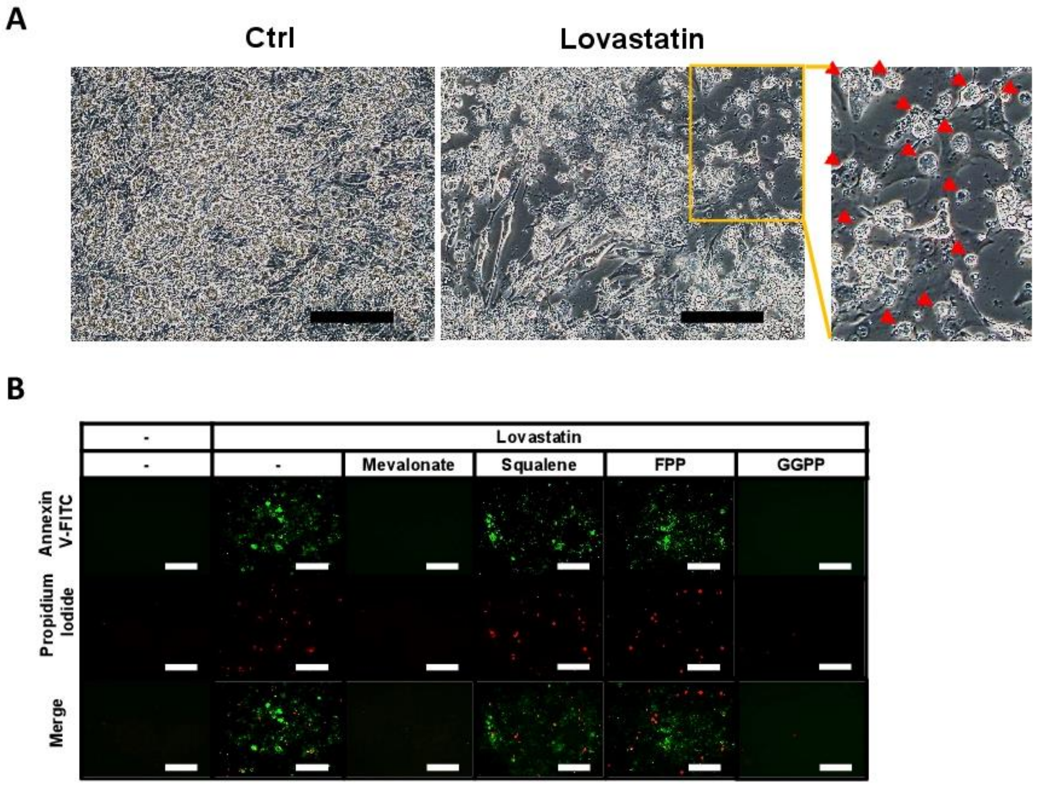

Figure S9. Lova Treatment Induces Apoptosis in Differentiated 3T3-L1 Adipocytes, Related to Figure 6

(A-B) After 3T3-L1 cells were differentiated and kept until D4, they were treated with 10 μM Lova in combination with or without MVA metabolites for an additional 4 days. Morphological observation of HMGCR-inhibited 3T3-L1 cells. Arrows indicate shrunken adipocytes (A). Annexin V (top panel) and PI (middle panel) staining was performed in differentiated 3T3-L1 adipocytes (B). Scale bars represent 200 μm. MVA metabolites included 30 μM MVA, 30 μM squalene, 10 μM FPP, and 10 μM GGPP.

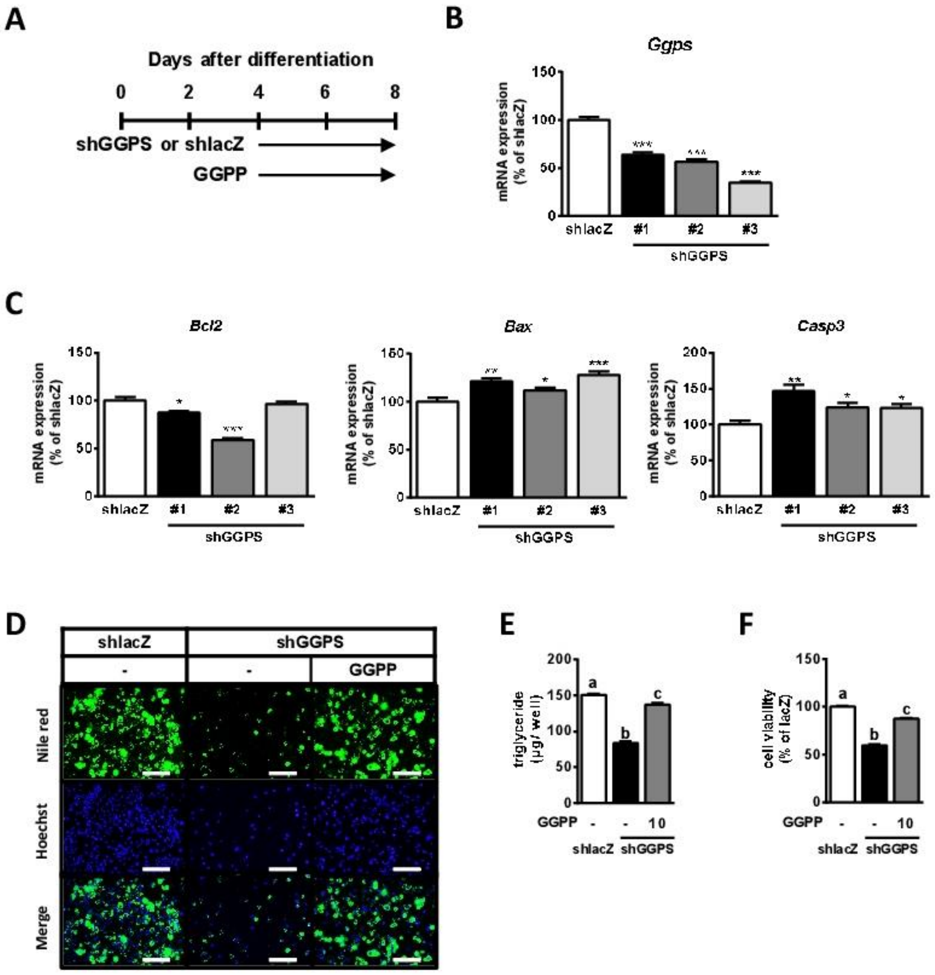

Figure S10. Knockdown of GGPP Synthase Induces Adipocyte Death, Related to Figure 7

(A-C) After coxsackie-adenovirus receptor (CAR)-overexpressed 3T3-L1 (CAR-L1) cells were differentiated and kept until D4, they were infected with adenovirus encoding *Ggps*-specific short hairpin RNA (shGGPS) or with control adenovirus encoding shRNA for lacZ gene in combination with or without GGPP for an additional 4 days (A). *Ggps* mRNA levels (B). Apoptosis-related gene expression (C).

(D-F) Differentiated CAR-L1 cells were infected with shGGPS#2 or shlacZ adenovirus in combination with or without 10 μM GGPP for an additional 4 days. The lipids and nuclei in CAR-L1 cells were stained with Nile red (top panel) and Hoechst33342 (middle panel), respectively (D). TG accumulation levels (E) and cell viability (F) are also shown. Scale bars represent 200 μm.

For detecting mRNA expression levels			
Gene	Forward primer	Reverse primer	Gene ID
Acox1	ACCTTCACTTGGGCATGTTC	TTCCAAGCCTCGAAGATGAG	11430
Acta2	GTACCACCATGTACCCAGGC	GCTGGAAGGTAGACAGCGAA	11475
Adgre1	TTTCCTCGCCTGCTTCTT C	CCCCGTCTCTGTATTCAACC	13733
Adipoq	TACAACCAACAGAATCATTATGACGG	GAAAGCCAGTAAATGTAGAGTCGTTGA	11450
Bax	GAGCTGCAGAGGATGATTGC	CTTGGATCCAGACAAGCAGC	12028
Bcl2	GTCGCTACCGTCGTGACTTC	CTGGGGCCATATAGTTCCACAA	12043
Casp3	GGAGCTTGGAACGGTACGC	CACATCCGTACCAGAGCGAG	12367
Col1a1	TTCAGCTTTGTGGACCTCCG	GGACCCTTAGGCCATTGTGT	12842
Cpt1a	CTCAGTGGGAGCGACTCTTCA	GGCCTCTGTGGTACACGACAA	12894
Fasn	AGGACTTGGGTGCTGACTACA	GGGAGCTATGGATGATGTTGA	14104
Fbp1	GGACTTTGACCCTGCCATCA	GGTGCCTTCTGGTGGATCTC	14121
Fn1	GTGGCTGCCTTCAACTTCTC	GTGGGTTGCAAACCTTCAAT	14268
G6pc	GCTGGAGTCTTGTCAGGCAT	ATCCAAGCGCGAAACCAAAC	14377
Ggps	CACTAGTGGCTTTAGTCAAGC	GAGTGTCTGAAGACAGCTAC	14593
Hmgcr	GGAGGCCTTTGATAGCACCA	TTCAGCAGTGCTTTCTCCGT	15357
Itgax	TGGGGTTTGTTTCTTGTCTTG	GCCTGTGTGATCGCCACATTT	16411
Pck1	AGGAGGAGTACGGGCAGTTG	CTTCAGCTTGCGGATGACA	18534
Ppara	TCGCGTACGGCAATGGCTTT	CTCTTCATCCCCAAGCGTAGGAGG	19013
Pparg	GGAGATCTCCAGTGATATCGACCA	ACGGCTTCTACGGATCGAAACT	19016
Rplp0	TCCTTCTTCCAGGCTTTGGG	GACACCCTCCAGAAAGCGAG	11837
Srebf1	GGAGCCATGGATTGCACATT	GCCAGAGAAGCAGAAGAGAAG	20787
Tgfb1	GCAACATGTGGAACTCTACCA	ACGTCAAAAGACAGCCACTCA	21803
Tnf	ACATCAGATCATCTTCTCAAAATTC	GTGTGGGTGAGGAGCACGTAGT	21926
For analyzing DNA allele			
Hmgcr	TTACTGGCTTGCTCAGCTTGCTCCA	GACACATGAAGGCATTCTCAGGCAT	15357

Table S1. Primers obtained for PCR amplification, Related to Figure 2, 4, 5, 6 and 7

	Ctrl	KO
BW (g)	33.94±0.93	39.45±0.73 ***
iWAT (mg)	0.41±0.07	0.05±0.003 **
eWAT (mg)	0.97±0.19	0.01±0.001 **
BAT (mg)	0.09±0.01	0.01±0.001 ***
Liver (mg)	1.36±0.07	5.21±0.33 ***
Heart (mg)	0.14±0.01	0.19±0.01 ***
Kidney (mg)	0.38±0.02	0.58±0.03 ***
Gastrocnemius Muscle (mg)	0.32±0.01	0.33±0.01

Table S2. Organs weight of male mice deleted HMGCR in adipose tissue, Related to Figure 1 and 2

Transparent Methods

Reagents

Mevalonic acid, IPP, FPP, GGPP and zoledronate were purchased from Sigma (St. Louis, MO, USA). Lovastatin, tamoxifen, and pioglitazone were obtained from Tokyo Kasei (Tokyo, Japan). Other chemicals were purchased from Sigma, Nacalai Tesque (Kyoto, Japan), or Wako Pure Chemicals (Osaka, Japan).

Animals

All animal experiments and maintenance were in accordance with the guidelines of the Kyoto University Animal Care Committee (approval code 28-49). Five-week-old C57BL/6 male mice were purchased from Japan SLC (Hamamatsu, Japan). All mice were housed at 23 ± 1 °C and maintained on a 12-h light/dark cycle. In all of the experiments, mice were fed a commercial chow diet (CRF-1, Charles River Japan, Kanagawa, Japan) until 8 weeks of age, and then the mice were randomly divided into groups for experiments.

Cell lines

3T3-L1 murine preadipocytes were cultured in Dulbecco's modified Eagle's medium supplemented with 10% fetal bovine serum and 100 U/ml penicillin as the growth medium at 37°C in 5% CO_2.

Primary adipose cells were prepared as in a previous study (Aune et al., 2013). Primary adipose cells were isolated from WAT of male HMGCR f/f mice and infected with ERT2-Cre expression retrovirus, which was kindly provided by Prof. Yasutomi Kamei (Kyoto Prefectural University, Kyoto, Japan). After 24 h of infection, cells were selected by 2.5 µg/ml puromycin for 1 week and used for cell culture experiments.

Mouse Phenotype Assessment

For examining the influence of adipose-specific HMGCR on metabolic state, the mice were fed a chow diet and sacrificed at 32 weeks of age. In the high fat diet (HFD) treatment experiment, mice were fed HFD containing 60% kcal fat (HFD, Research Diet, New Brunswick, NJ, USA) for 20 weeks. Pio treatment was administered for 10 weeks. In

the high cholesterol diet treatment experiment, 18-week-old mice were fed the control diet (AIN-93M, Oriental Yeast Co., Tokyo, Japan) or AIN-93M, containing 1% (w/w) of cholesterol, for 6 weeks. To perform the GTT assay, mice were fasted for 6 h and a blood sample was taken from the mouse tail vein (0 min point). Subsequently, mice were orally treated with glucose solution (2 g of glucose/ 10 ml of sterile phosphate-buffered saline, 10 μl/g body weight) and blood samples were collected at different time points (15, 30, 60, and 120 min). For analysis of ITT, blood samples were collected from the mouse tail vein (0 min point) and the mice were then intraperitoneally injected with insulin solution (0.1 U of insulin/ml of sterile phosphate-buffered saline, 10 μl/g body weight) after fasting for 6 h. The blood samples were taken at 15, 30, 60, and 120 min. The oxygen consumption was measured using an indirect calorimetric system (Oxymax Equal Flow 8 Chamber/Small Subject System; Columbus Instruments, Columbus, OH, USA) equipped with an eight-chamber airtight metabolic cage. The RER was calculated by dividing the CO_2 production by the O_2 consumption. The locomotor activity was measured using an Actimo-S system (Bio Research Centre, Nagoya, Japan). For both oxygen consumption and locomotor activity measurement, mice were acclimatized to the individual experiment-specific cages for 2 hours prior to the experiment and measurement was performed from 15:00 (ZT8) to the next day at 11:00 (ZT4) in a 12-h light/dark cycle condition. Body composition was scanned using an EchoMRI 3-in-1 system (Hitachi, Tokyo, Japan), in accordance with the manufacturer's instructions.

Strategy and Generation of aKO mice

The floxed HMGCR mice (HMGCR f/f) were generated by Unitech Co. (Chiba, Japan). Adipoq-Cre BAC transgenic mice (RRID: IMSR_JAX:010803) were purchased from Jackson Laboratory (Bar Harbor, ME, USA). To generate floxed HMGCR mice, loxP sites were introduced on either side of exons 12 and 14 of *Hmgcr*. Adipoq-Cre BAC transgenic mice, in which Cre recombinase is specifically expressed in adipose tissue, were used to cross floxed HMGCR mice (HMGCR f/f) (Figure S1A) to generate HMGCR f/+ Cre+ progeny. These mice were then backcrossed to HMGCR f/f mice. The backcrossed HMGCR f/+ Cre+ mice were subsequently interbred to yield four derivative strains: (i) HMGCR f/f (Ctrl), (ii) HMGCR f/f Cre+ (aKO), (iii) HMGCR f/+ Cre+ (heterogeneous aKO), and (iv) HMGCR f/+. aKO mice were predicted to have adipose-specific loss of *Hmgcr* exons 12–14, which was expected to abolish translation of the entire carbohydroxylation site (Asn 281) containing the catalytic activity (Liscum et al., 1985).

Adipose Tissue Implantation

The adipose tissue implantation was performed as previous study (Gavrilova et al., 2000). To prevent rejection, donors and recipients were sex-matched. Five-week-old C57BL/6J mice were used as fat donors. Donor adipose tissues from euthanized C57BL/6J mice were placed into sterile phosphate-buffered saline and cut into 100- to 150-mg pieces. The fat grafts were implanted subcutaneously in the shaved skin of the backs of anesthetized 8-week-old aKO mice via small incisions. Into two incisions, 3 to 4 fat grafts were placed, totaling 1000 mg of fat per mouse. After surgery, mice were housed individually for 2 weeks.

Cell culture

Two days after reaching confluence (D0), 3T3-L1 cells were incubated in a differentiation medium containing 0.25 μM dexamethazone, 10 μg/ml insulin, and 0.5 mM 3-isobutyl-1-methylxanthine in the growth medium. After 48 h (D2), the cell culture medium was changed to post differentiation medium containing 5 μg/ml insulin in the growth medium, and a fresh post-differentiation medium was also supplied at Day 4 (D4). At 4 days after differentiation, 3T3-L1 cells were used for subsequent experiments. To increase the efficiency of adenovirus-regulated transduction of short hairpin RNA (shRNA), 3T3-L1 adipocytes stably expressing coxsackie-adenovirus receptor (CAR)-L1 were obtained (Ito et al., 2007). Four days after differentiation, CAR-L1 cells were re-seeded into a new plate and infected with adenovirus encoding *Ggps*-specific shRNA or with control adenovirus encoding lacZ, for additional 4 days. The target sequences were as follows: Ggps#1, 5′-GGAACCGTCAGCTTTGAAATTTA-3′; Ggps#2, 5′-GTCCAACTGAAGAAGAATATAAA-3′; Ggps#3, 5′-CAGCTGTTCTCTGATTACAAAGA-3′; lacZ control, 5′-GCTACACAAATCAGCGATTT-3′.

ERT2-Cre-infected HMGCR f/f primary WAT cells were differentiated by a method consistent with that used for 3T3-L1 cells. For lipid staining, the adipocytes were double-stained with 10 μg/ml Nile red and 20 μM Hoechst 33324. For apoptosis staining, the Annexin V-FITC Apoptosis Detection Kit (Nacalai Tesque) was used in accordance with the manufacturer's instructions.

Histological Analysis

The organ samples of BAT, iWAT, eWAT, and the liver were fixed in 4% paraformaldehyde. The fixed samples were

embedded in paraffin for staining with eosin Y (Wako Pure Chemicals) or embedded in O.C.T. compound (Sakura Finetek Japan, Tokyo, Japan) for staining with Oil Red O. Both eosin Y- and Oil Red O-stained cells were counterstained with modified Mayer's hematoxylin (Merck, Darmstadt, Germany).

Hepatic Lipid Analysis

To quantify hepatic TG content, the lipids were extracted by 1.2 ml of hexane/2-propanol (3:2 v/v) for each 50 mg of liver samples. The liver samples were homogenized and centrifuged at 4 °C for 10 min at 10,000 ×*g*. After drying by evaporation, the lipid extracts were resuspended in 2-propanol and determined enzymatically using the TG E-test (Wako Pure Chemicals).

Hepatic Glycogen Analysis

Eppendorf tubes containing 600 µl of 30% (W/V) KOH were used to extract 100 mg of each liver sample, followed by incubation at 100 °C until the sample dissolved. Then, 100 µl of saturated Na_2SO_4 and 750 µl of 95% EtOH were added and vortexed, and then incubated at 100°C until small bubbles were confirmed. The samples were centrifuged at 4°C for 10 min at 300 ×*g*, and the supernatant was decanted to dry the samples. Then, 400 µl of distilled water and 500 µl of 95% EtOH were added to all samples and mixed. After incubation at 100°C until small bubbles formed, the samples were centrifuged at 4°C and 300 ×*g* for 10 min. The samples were then decanted and dried, and 1.2 ml of 0.6 N HCl was added with incubation at 100 °C for 2 to 2.5 hours. From each sample, 300 µl was removed to a new Eppendorf tube containing 415 µl of 0.5 N NaOH and 285 µl of distilled water and mixed. The concentration of the samples was measured by glucose C-test.

Analysis of Plasma Chemical Parameters

The levels of glucose, TG, cholesterol, β-HB, FFA, GOT/GPT, leptin, insulin, and adiponectin were determined using commercially available kits: glucose C-test, triacylglycerol E-test, cholesterol E-test, autokit 3-HB, NEFA C-test, transaminase C-test (Wako Pure Chemicals), mouse/rat leptin ELISA kit, ultrasensitive mouse insulin assay kit (Morinaga Institute of Biologic Science, Yokohama, Japan), and mouse adiponectin/Acrp30 Quantikine ELISA kit

(R&D Systems, Minneapolis, MN, USA), respectively. All kits were used in accordance with the manufacturer's instructions.

Gene Expression Quantification

RNA was isolated with Sepasol-RNA I Super reagent (Nacalai Tesque) and then reverse-transcribed using M-MLV reverse transcriptase (Promega Corporation, Fitchburg, WI, USA). For quantifying the mRNA expression level, real-time PCR was performed using a LightCycler system (Roche Diagnostics, Mannheim, Germany) using SYBR Green fluorescence signals (AptaTaq DNA Polymerase). The protocol for amplification was as follows: denaturation, 95°C for 1 min; annealing, 60°C for 5 sec; extension, 72°C for 30 sec. All measured gene expression was normalized to the levels of ribosomal protein lateral stalk subunit P0 (*Rplp0*). Primer sequences are provided in Table S1.

DNA Extraction and Detection

The genomic DNA of tissue and cell samples was extracted using 100 µl of Proteinase K solution (50 mM Tris-HCl buffer pH 8.0, 25 mM EDTA, 0.5% SDS, 100 mM NaCl and Proteinase K 1 mg/ml) for incubation at 50°C overnight. After the samples were dissolved, 100 µl of sterile water and 200 µl of phenol:chloroform:isoamyl alcohol (25:24:1) were added and gently mixed. The samples were then centrifuged at 4°C for 5 min at 14,000 ×*g*, and 150 µl of supernatant was removed into a new Eppendorf tube and then 200 µl of chloroform was added to every sample. After centrifugation at 4°C for 5 min at 14,000 ×*g*, 120 µl of supernatant was collected and removed into an Eppendorf tube containing 16 µl of 3M Na-acetate and 400 µl of 99.5% ethanol. The DNA pellet was precipitated through centrifugation at 4°C for 20 min and 19,000 ×*g*. After the supernatant was discarded, the DNA pellet was dissolved immediately in 30 µl of TE buffer (pH 8.0). The genomic DNA was amplified using EmeraldAmp PCR Master Mix and confirmed by agarose gel electrophoresis (Watanabe et al., 2015). Primer sequences are listed in Table S1.

QUANTIFICATION AND STATISTICAL ANALYSIS

All of the results are given as the mean ± SEM. Bars in graphs represent standard errors, and significance was assessed by Student's two-tailed *t* tests or one-way analysis of variance with a post-hoc Tukey HSD test. Differences

with $p < 0.05$ were considered statistically significant.

Supplemental References

Aune, U.L., Ruiz, L., and Kajimura, S. (2013). Isolation and differentiation of stromal vascular cells to beige/brite cells. Journal of visualized experiments : JoVE.

Gavrilova, O., Marcus-Samuels, B., Graham, D., Kim, J.K., Shulman, G.I., Castle, A.L., Vinson, C., Eckhaus, M., and Reitman, M.L. (2000). Surgical implantation of adipose tissue reverses diabetes in lipoatrophic mice. The Journal of clinical investigation *105*, 271-278.

Ito, A., Suganami, T., Miyamoto, Y., Yoshimasa, Y., Takeya, M., Kamei, Y., and Ogawa, Y. (2007). Role of MAPK phosphatase-1 in the induction of monocyte chemoattractant protein-1 during the course of adipocyte hypertrophy. The Journal of biological chemistry *282*, 25445-25452.

Liscum, L., Finer-Moore, J., Stroud, R.M., Luskey, K.L., Brown, M.S., and Goldstein, J.L. (1985). Domain structure of 3-hydroxy-3-methylglutaryl coenzyme A reductase, a glycoprotein of the endoplasmic reticulum. The Journal of biological chemistry *260*, 522-530.

Watanabe, M., Suzuki, Y., Uchida, K., Miyazaki, N., Murata, K., Matsumoto, S., Kakizaki, H., and Tominaga, M. (2015). Trpm7 Protein Contributes to Intercellular Junction Formation in Mouse Urothelium. The Journal of biological chemistry *290*, 29882-29892.

Yeh, Y.S., Goto, T., Takahashi, N., Egawa, K., Takahashi, H., Jheng, H.F., Kim, Y.I., and Kawada, T. (2016). Geranylgeranyl pyrophosphate performs as an endogenous regulator of adipocyte function via suppressing the LXR pathway. Biochemical and biophysical research communications *478*, 1317-1322.
